# Deciphering Physio-Biochemical Basis of Tolerance Mechanism for Sesame (*Sesamum indicum* L.) Genotypes under Waterlogging Stress at Early Vegetative Stage

**DOI:** 10.3390/plants13040501

**Published:** 2024-02-10

**Authors:** Vishal Chugh, Vigya Mishra, Vijay Sharma, Mukul Kumar, Mouna Ghorbel, Hitesh Kumar, Ashutosh Rai, Rahul Kumar

**Affiliations:** 1 Department of Basic & Social Sciences, College of Horticulture, Banda University of Agriculture and Technology, Banda 210001, India; vishalchugh3@gmail.com; 2 Department of Postharvest Technology, College of Horticulture, Banda University of Agriculture and Technology, Banda 210001, India; vigyamishra@buat.edu.in; 3 Department of Genetics & Plant Breeding, College of Agriculture, Banda University of Agriculture and Technology, Banda 210001, India; mukulkumar@buat.edu.in (M.K.); hiteshkumar@buat.edu.in (H.K.); 4 Biology Department, Faculty of Science, University of Hail, Ha’il P.O. Box 2440, Saudi Arabia; m.ghorbel@uoh.edu.sa; 5 ORISE Participant Sponsored by the U.S. Vegetable Laboratory, USDA ARS, 2700 Savannah Highway, Charleston, SC 29414, USA

**Keywords:** waterlogging, sesame, reactive oxygen species, ethanolic fermentation, antioxidant enzymes

## Abstract

Waterlogging represents a substantial agricultural concern, inducing harmful impacts on crop development and productivity. In the present study, 142 diverse sesame genotypes were examined during the early vegetative phase to assess their response under waterlogging conditions. Based on the severity of symptoms observed, 2 genotypes were classified as highly tolerant, 66 as moderately tolerant, 69 as susceptible, and 5 as highly susceptible. Subsequent investigation focused on four genotypes, i.e., two highly tolerant (JLT-8 and GP-70) and two highly susceptible (R-III-F6 and EC-335003). These genotypes were subjected to incremental stress periods (0 h, 24 h, 48 h, 72 h, and 96 h) to elucidate the biochemical basis of tolerance mechanisms. Each experiment was conducted as a randomized split-plot design with three replications, and the statistical significance of the treatment differences was determined using the one-way analysis of variance (ANOVA) followed by the Fisher least significant difference (LSD) test at *p ≤* 0.05. The influence of waterlogging stress on morphological growth was detrimental for both tolerant and susceptible genotypes, with more severe consequences observed in the latter. Although adventitious roots were observed in both sets of genotypes above flooding levels, the tolerant genotypes exhibited a more rapid and vigorous development of these roots after 48 h of stress exposure. Tolerant genotypes displayed higher tolerance coefficients compared to susceptible genotypes. Furthermore, tolerant genotypes maintained elevated antioxidant potential, thereby minimizing oxidative stress. Conversely, susceptible genotypes exhibited higher accumulation of hydrogen peroxide (H_2_O_2_) and malondialdehyde content. Photosynthetic efficiency was reduced in all genotypes after 24 h of stress treatment, with a particularly drastic reduction in susceptible genotypes compared to their tolerant counterparts. Tolerant genotypes exhibited significantly higher activities of anaerobic metabolism enzymes, enabling prolonged survival under waterlogging conditions. Increase in proline content was observed in all the genotypes indicating the cellular osmotic balance adjustments in response to stress exposure. Consequently, the robust antioxidant potential and efficient anaerobic metabolism observed in the tolerant genotypes served as key mechanisms enabling their resilience to short-term waterlogging exposure. These findings underscore the promising potential of specific sesame genotypes in enhancing crop resilience against waterlogging stress, offering valuable insights for agricultural practices and breeding programs.

## 1. Introduction

The abiotic challenge of waterlogging stress has emerged as a substantial threat to worldwide agricultural productivity. This phenomenon is exacerbated by unpredictable precipitation patterns and severe climatic events, posing a significant risk to global crop cultivation [1]. Most land plants, including crops, rely on aerobic conditions, consequently, waterlogged habitats significantly impede plant growth and productivity by imposing constraints on, or complete deprivation of, crucial oxygen supply [2]. Waterlogging precipitates a myriad of adverse effects on plants, including chlorosis, leaf senescence, browning, wilting, necrosis, stunted growth, modified root permeability, flower abortion, diminished hydraulic conductivity, delayed maturity, and increased susceptibility to diseases and pests. These multifaceted impacts significantly hinder the overall development and growth of plants [3]. Under hypoxic conditions, especially anoxic ones, tissues block oxygen-dependent functions, which prevents the uptake of carbon and the use of photosynthetic energy. This disruption in functional linkages, especially concerning the internal movement of oxygen from roots to shoots, exacerbates the existing challenges [4,5]. Under waterlogging conditions, plants initiate a spectrum of physiological, metabolic, biochemical, and mitigation mechanisms to ensure survival and facilitate growth. The reaction of the plant to waterlogging commences with the initiation of signal transduction components, initiating metabolic adaptations, notably fermentation metabolism. This process ultimately results in morphological alterations, including the development of aerenchyma and adventitious roots, contingent upon the plant’s resilience level [2,6].

Facilitating the biosynthesis of compatible solutes represents a key biochemical adaptation aimed at augmenting stress tolerance in plants. In order to preserve osmotic equilibrium, plants accumulate a diverse array of non-harmful solutes termed as compatible solutes. These include trehalose, fructans, polyols, proline, glycinebetaine, and polyamines, all of which exert no disruptive effects on vital plant processes. Notably, proline accumulation is the primary response of plants to stress, and it has been suggested to play a major role in stability of membranes, proteins, and subcellular structures [7,8].

Waterlogging-induced hypoxia disrupts plant root respiration, leading to decreased energy levels within root cells. This impairment negatively affects vital cellular processes like the Tricarboxylic acid cycle and Electron transport chain due to the scarcity of oxygen, which serves as the terminal acceptor for electrons in aerobic respiration. Consequently, plants experiencing waterlogging adapt by shifting to a less efficient fermentative metabolism to generate ATP [5,9,10]. This metabolic adaptation triggers the synthesis of anaerobic proteins (ANPs), notably alcohol dehydrogenase (ADH), pyruvate decarboxylase (PDC), aldehyde dehydrogenase (ALDH), and lactate dehydrogenase (LDH). These proteins are essential for plant survival under oxygen-deprived conditions. During flooding-induced stress, Pyruvate is converted by PDC to acetaldehyde which is subsequently metabolized into ethanol through ADH. This enzymatic process ensures the nicotinamide adenine dinucleotide (NAD^+^) regeneration, thereby sustaining glycolytic pathways. Genotypic variants lacking functional PDC and ADH enzymes underscore the essential role of ethanol fermentation in adapting to the stress conditions induced by flooding [11]. The generation of ethanol exhibits harmless characteristics due to its swift extracellular diffusion from cells, whereas the intermediary acetaldehyde exerts toxicity. Acetaldehyde is enzymatically transformed into acetate by ALDH, concurrently facilitating the reduction of NAD^+^ to NADH [12].

Waterlogging-induced plant damage results in decreased photosynthetic efficiency, primarily due to inhibited activity of vital enzymes like ribulose 1,5-bisphosphate carboxylase, glycolate oxidase, and phosphoglycolate, along with damage to chloroplast membranes [13]. This photosynthetic impairment, triggered by waterlogging stress, elevates the production of deleterious reactive oxygen species (ROS), including singlet oxygen (^1^O_2_), superoxide (O_2_^•−^), hydroxyl radicals (OH^•^), and hydrogen peroxide (H_2_O_2_) [14]. In response to the damaging effects of ROS, plants employ a variety of protective mechanisms. These mechanisms encompass enzymatic factors like superoxide dismutase (SOD), catalase (CAT), glutathione reductase (GR), ascorbate peroxidase (APX), peroxidase (POX), monodehydroascorbate reductase (MDHAR), and dehydroascorbate reductase (DHAR). Additionally, non-enzymatic components like ascorbate (AsA), reduced glutathione (GSH), carotenoids, and tocopherols play a vital role in safeguarding plants’ lipids and other essential molecules from oxidative damage [15,16]. These protective traits are essential for waterlogging-resilient plants. It has been reported that the elevated activities of SOD, CAT, APX, and GR are indispensable for the survival of mungbean [17], rice [18], maize [12], sunflower [19], and wheat [15] under waterlogging conditions. The accrual of H_2_O_2_ and malondialdehyde (MDA), signifying lipid peroxidation, it has been proposed, acts as a universal marker for tolerance to waterlogging stress [20].

Sesame (*Sesamum indicum* L.), an erect annual herb commonly known as sesamum, benniseed, or simsim, represents one of the earliest and most valuable oilseed crops, esteemed for its high-quality seed oil. Taxonomically, it falls under the Pedaliaceae family. India, China, and Burma stand as the foremost global producers of sesame, with its cultivation offering substantial economic advantages to farmers in these regions. Despite being recognized as a drought-resistant oil crop, sesame exhibits a notable vulnerability to flooding events [21]. In instances where sesame crops are cultivated in soil exhibiting inadequate drainage characteristics, the production undergoes detrimental consequences attributable to recurrent and intense precipitation, which results in substantial yield reductions, surpassing 30% and, in severe scenarios, ranging from 50 to 90% [22,23]. Field studies have demonstrated that under waterlogged conditions, sesame crops experience premature senescence due to leaf chlorosis, defoliation, necrosis, and impaired nitrogen fixation, resulting in a cessation of growth and decreased yields [23]. It has been substantiated that there exists genetic diversity concerning the trait of tolerance to waterlogging [24,25]. Several research studies have established that sesame plants, when subjected to waterlogging stress, elicit diverse morpho-physiological, anatomical, and biochemical responses [21,22,23]; however, very few studies have explored the biochemical mechanism with respect to anaerobic metabolism and the antioxidant system alongside the morph-anatomic adaptations in sesame under waterlogged conditions. In order to comprehend the synchronized actions of antioxidant defense and anaerobic metabolism in conferring waterlogging stress tolerance in sesame, the authors first screened 142 lines of sesame against waterlogging stress at the vegetative stage. Following this, four contrasting genotypes, i.e., two highly tolerant and two highly susceptible genotypes, were subjected to biochemical and physiological studies against incremental waterlogging stress.

## 2. Results

### 2.1. Screening Experiment

**Figure 1:**
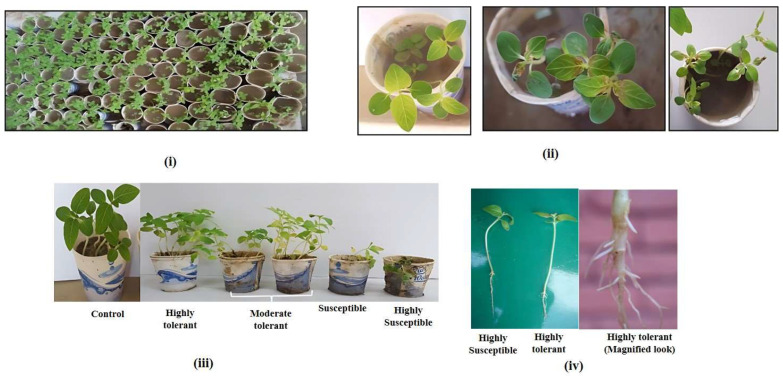
(**i**) Screening experiment set up; (**ii**) waterlogging stress-induced symptoms showing yellowing, browning, and wilting of leaves used as phenotypic markers for screening; (**iii**) phenotypic status of plants under different categories at the end of the experiment; and (**iv**) adventitious roots formation in tolerant and susceptible genotypes.

At the seedling stage, 142 diverse sesame genotypes were screened for waterlogging stress tolerance to identify tolerant and susceptible genotypes (Figure 1i). The appearance of symptoms like leaf yellowing (chlorosis), leaf browning, drooping, wilting, and leaf necrosis were selected as the parameters for the stress-induced injury in the plants (Figure 1ii–1iii). The observations were recorded for 7 consecutive days. Remarkably, all the sesame genotypes exhibited varying degrees of stress-induced injury, albeit with differing onset and severity levels. On the basis of the appearance and severity of the symptoms, we could identify 2 as highly tolerant, 66 as moderately tolerant, 69 as susceptible, and 5 as highly susceptible genotypes. Notably, the highly susceptible genotypes experienced nearly 100% mortality due to the stress conditions.

### 2.2. Morpho-Physiological and Biochemical Characterization of Selected Genotypes

Based on the above screening results, four genotypes with constating response towards waterlogging (two highly tolerant and two highly susceptible) were further subjected to morphological and biochemical characterization under incremental stress treatments.

#### 2.2.1. Morpho-Physiological Characters

**Figure 2:**
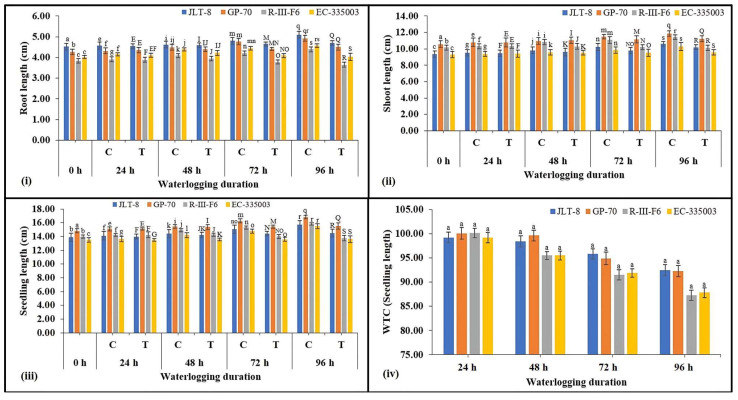
Impact of incremental waterlogging stress on the activities of (**i**) root length, (**ii**) shoot length, (**iii**) seedling length, and (**iv**) WTC (seedling length) in tolerant and susceptible sesame genotypes. Values are mean ± SD of three replicates. Different letters depict significant differences between the genotypes at *p ≤* 0.05 (Fisher LSD test).

**Figure 3:**
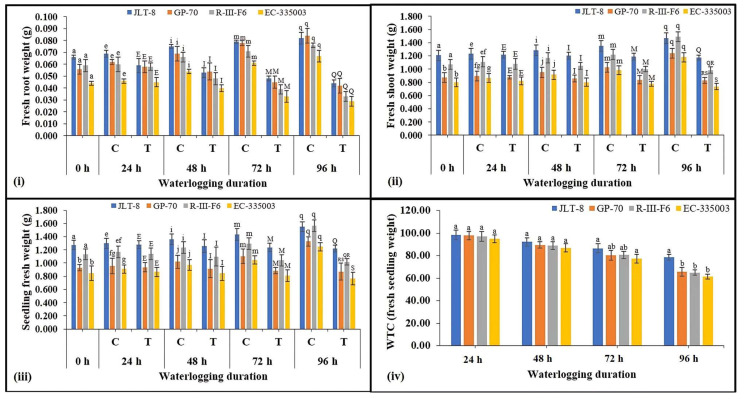
Impact of incremental waterlogging stress on the activities of (**i**) fresh root weight, (**ii**) fresh shoot weight, (**iii**) fresh seedling weight, and (**iv**) WTC (fresh seedling weight) in tolerant and susceptible sesame genotypes. Values are mean ± SD of three replicates. Different letters depict significant differences between the genotypes at *p ≤* 0.05 (Fisher LSD test).

**Figure 4:**
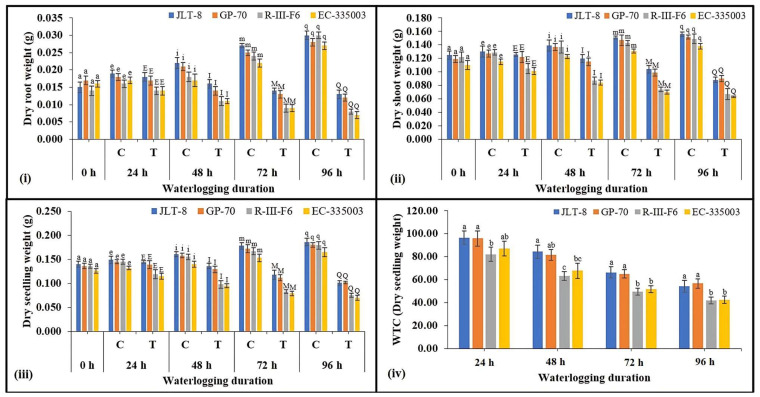
Impact of incremental waterlogging stress on the activities of (**i**) dry root weight, (**ii**) fresh dry shoot weight, (**iii**) dry seedling weight, and (**iv**) WTC (dry seedling weight) in tolerant and susceptible sesame genotypes. Values are mean ± SD of three replicates. Different letters depict significant differences between the genotypes at *p ≤* 0.05 (Fisher LSD test).

All the genotypes exhibited the typical physiological and morphological consequence to waterlogging stress. Increasing the duration of waterlogging stress caused a reduction in root length compared to their respective controls, in tolerant as well as susceptible genotypes (Figure 2i), although it is evident that tolerant genotypes maintained higher root length as compared to susceptible genotypes at all the stressed conditions. A similar pattern of results was also found in terms of shoot length (Figure 2ii); however, the decrease in genotypes was not very significant. Total seedling length was decreased in both groups of genotypes under waterlogging stress imposed for 48 h and above (Figure 2iii). Where susceptible genotypes showed a moderate decrease of 13% (R-III-F6) and 12% (EC-335003) compared to their respective controls, tolerant genotypes exhibited a very non-significant decrease under all treatments. A very moderate difference in WTC on the basis of seedling length was observed between tolerant and susceptible genotypes. Under the highest studied treatment (96 h), tolerant genotypes showed WTC (seedling length) in the range of 92% while susceptible genotypes showed a WTC of 87% (Figure 2iv). Results obtained from fresh root weight indicate severe loss in tolerant as well as susceptible genotypes due to waterlogging stress (Figure 3i). Maximum stress treatment, i.e., 96 h of stress, caused a significant decrease of 46 and 50% in JLT-8 and GP-70, and 57 % in R-III-F6 and EC-335003 (Figure 3i). Similar loss was also observed in fresh shoot weight of plants under study (Figure 3ii). Waterlogging stress led to 20–33% loss in fresh shoot weight of tolerant genotypes and 34–38% loss in susceptible genotypes at the highest stress treatment in comparison to their respective controls at same stage. Seedling fresh weight also declined with increasing stress intensity in both groups of genotypes. Tolerant genotypes exhibited a reduction in fresh seedling weight in the range of 21.5–34.4%, while a decrease in the range of 35–39% was noted for susceptible genotypes after 96 h of waterlogging stress imposition (Figure 3iii). WTC was calculated on the basis of fresh seedling weight among the tolerant genotypes; JLT-8 showed the highest WTC of 78.49% while other all genotypes showed WTC in the range of 61–65% at highest waterlogging stress treatment (Figure 3iv). A substantial decrease in dry root and shoot weight was observed in all the genotypes in response to increasing waterlogging stress (Figure 4i,ii). However, while both genotypes showed a marked decrease in their dry root and shoot weight, the loss was more significant in susceptible genotypes compared to tolerant genotypes. Where both tolerant genotypes exhibited a reduction of 57% in dry root weight and 44% (JLT-8) and 41% (GP-70) in dry shoot weight at the end of the experiment compared to their respective controls, the susceptible genotypes showed 73 (R-III-F6) and 74% (EC-335003) reduction in dry root weight and 55 (R-III-F6) and 54% (EC-335003) decrease in dry shoot weight after 96 h of waterlogging compared to the unstressed samples (Figure 4i,ii). Data also show that the dry seedling weight decreased in both groups of genotypes, but the drop was more severe in susceptible genotypes (58% for both the genotypes) than the tolerant genotypes (46% for JLT-8 and 43% for GP-70) at highest treatment conditions (Figure 4iii). WTC calculated on the basis of dry seedling weight revealed that tolerant genotypes exhibited higher WTC than susceptible genotypes at all stress conditions (Figure 4iv). The exposure of waterlogging stress caused the initiation of adventitious root formation in both tolerant and susceptible lines, above the flooding threshold. Nonetheless, in tolerant genotypes, the development was more rapid and vigorous, manifesting within 48 h of stress exposure. In contrast, susceptible genotypes displayed a delayed response, with adventitious root development becoming noticeable only after 72 h of stress exposure. (Figure 1iv).

#### 2.2.2. Biochemical characters

##### Antioxidant System

Status of antioxidant enzymes

**Figure 5:**
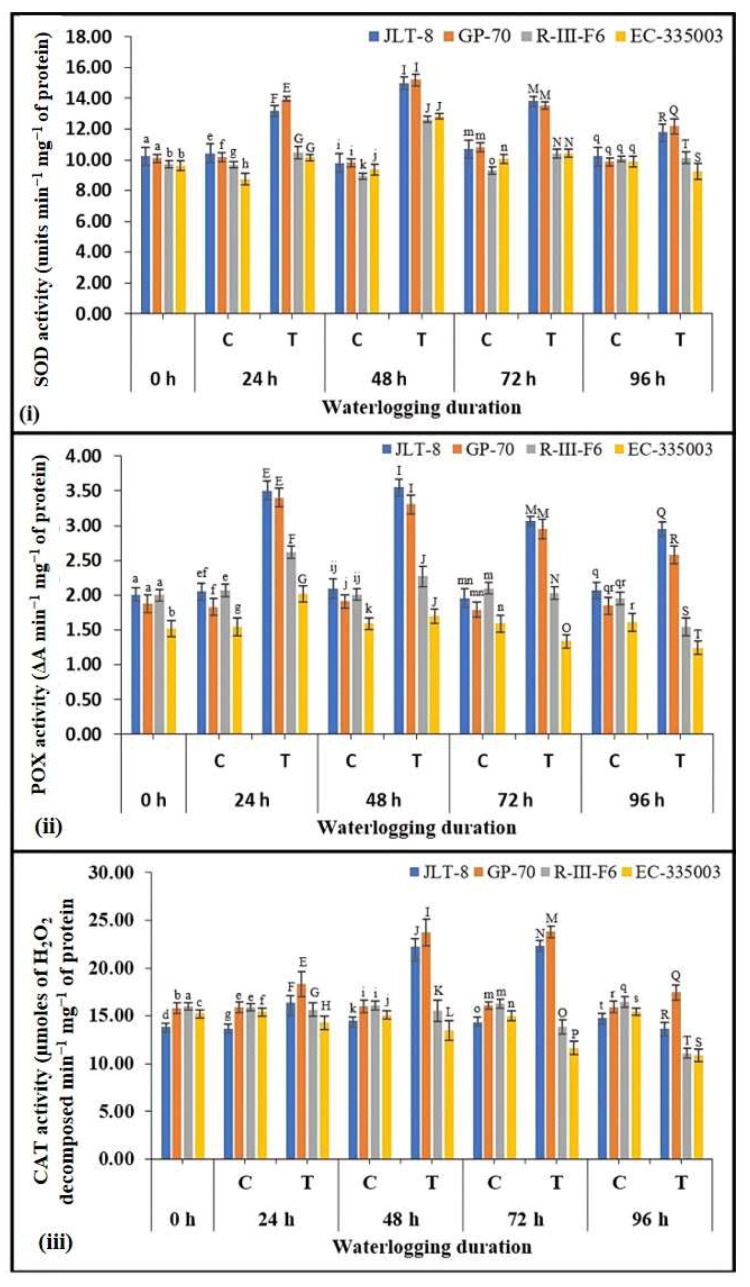
Impact of incremental waterlogging stress on the activities of (**i**) superoxide dismutase (SOD), (**ii**) peroxidase (POX), and (**iii**) Catalase (CAT) in tolerant and susceptible sesame genotypes. Values are mean ± SD of three replicates. Different letters depict significant differences between the genotypes at *p ≤* 0.05 (Fisher LSD test).

SOD transforms the O_2_^•−^ generated to H_2_O_2_ and O_2_. Specific activity of SOD was found to be significantly increased in leaf samples of tolerant as well as susceptible genotypes, with increasing intensity of stress till 48 h, after which activity decreased in all the genotypes (Figure 5i). However, the tolerant genotypes exhibited higher activity under all the conditions, with the highest activity at 14.99 units min^−1^ mg^−1^ of protein for JLT-8 and 15.20 units min^−1^ mg^−1^ of protein for GP-70, whereas susceptible genotypes showed the highest activity in the range of 12.63 (for R-III-F6) to 12.83 (for EC-335003) units min^−1^ mg^−1^ of protein. Even at maximum stress treatment, i.e., at 96 h, the tolerant genotypes exhibited higher SOD activity than the susceptible genotypes (Figure 5i).

A differential response was observed in terms of specific activity of POX between tolerant and susceptible genotypes (Figure 5ii). Tolerant genotypes exhibited significantly higher POX specific activity in leaves under stress conditions in comparison to the controls, while in susceptible genotypes the activity was found to be slightly higher till 48 h, after which a decrease in activity was observed when compared to the controls. Tolerant genotypes maintained significantly higher POX activity even at highest stress treatment regime (45.2% higher in JLT-8 and 39.5% higher in GP-70). Waterlogging stress resulted in a significant increase in POX activity in leaves of tolerant genotypes, while a moderate rise followed by decrease in activity was observed in susceptible genotypes (Figure 5ii).

Maintaining catalase is crucial when plants are exposed to extreme waterlogging conditions because it scavenges H_2_O_2_, which is primarily produced during photorespiration. Among the two groups of genotypes, CAT activity was found to be elevated in the leaves of both the tolerant genotypes till 72 h of stress treatment, achieving 55.5% and 48% rise in JLT-8 and GP-70, respectively, after a significant decline in activity was observed in 96 h stress treatment samples (Figure 5iii). In contrast, susceptible genotypes exhibited a consistent decrease in activity in all the stressed samples with respect to the controls. The activity was dropped by 33% (for R-III-F6) and 29% (for EC-335003) as compared to controls (Figure 5iii).

Halliwell–Asada cycle enzymes and their associated metabolites

**Figure 6:**
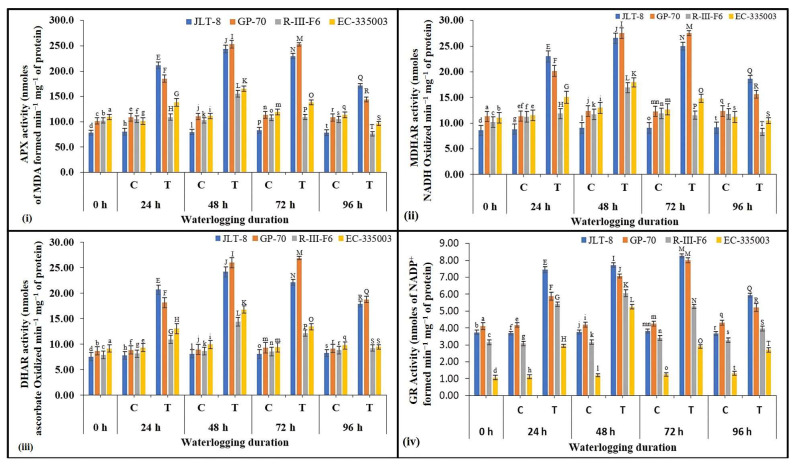
Impact of incremental waterlogging stress on the activities of ascorbate–glutathione cycle enzymes in tolerant and susceptible sesame genotypes: (**i**) ascorbate peroxidase (APX); (**ii**) monodehydroascorbate reductase (MDHAR); (**iii**) dehydroascorbate reductase (DHAR); and (**iv**) Glutathione reductase (GR). Values are mean ± SD of three replicates. Different letters depict significant differences between the genotypes at *p ≤* 0.05 (Fisher LSD test).

Using ascorbate as an electron donor, APX catalyzes the initial step of the AsA-GSH pathway and protects the cell by converting H_2_O_2_ to water. A similar pattern was observed for the APX activity in tolerant as well as susceptible genotypes in response waterlogging stress (Figure 6i). Both groups exhibited a significant increase in APX activity till 48 h of stress treatment, after which the activity was decreased significantly till 96 h, with the exception of GP-70 which was found to maintain activity at 72 h of stress duration followed by a steep decline in activity. It is worth noting that the increase in activity was substantially higher in tolerant genotypes (192.5% for JLT-8 and 55% for GP-70) compared to susceptible genotypes (44% for R-III-F6 and 38.5% for EC-335003) with respect to their controls (Figure 6i). Tolerant genotypes also maintained significantly higher APX activity (171.1 and 143.8 nmoles of MDA formed min^−1^ mg^−1^ of protein for JLT-8 and GP-70, respectively) even at the maximum stress treatment, i.e., at 96 h, than the susceptible genotypes (76.2 and 96.5 nmoles of MDA formed min^−1^ mg^−1^ of protein for R-III-F6 and EC-335003, respectively).

Waterlogging caused enhanced MDHAR activity in tolerant genotypes till 48 h of stress treatment, which was maintained till 72 h then followed by decrease in activity. A significant rise of 192.5% and 123% was recorded at its peak in JLT-8 and GP-70 compared to the unstressed controls (Figure 6ii). The MDHAR activity in susceptible genotypes was also found to be moderately increased till 48 h of stress (44% and 38.6% for R-III-F6 and EC-335003), after which activity decreased sharply (Figure 6ii). Tolerant genotypes maintained higher MDHAR activity under all stress conditions as compared to the susceptible genotypes.

A strong increase in DHAR activity was noted for tolerant genotypes in response to waterlogging stress, which reached a peak at 48 h and was sustained till 72 h, also followed by a significant decrease (Figure 6iii). The highest DHAR activity of 24.20 units (198% higher than the controls) for JLT-8 was recorded at 48 h of waterlogging stress, and 26.90 units (189% higher than the controls) for GP-70 at 72 h. A moderate rise in activity was observed in susceptible genotypes, reaching maximum activity after 48 h waterlogging stress (14.39 and 16.80 units for R-III-F6 and EC-335003, respectively). The activity was declined thereafter, on the exposure to further severe stress conditions. Tolerant genotypes were able to maintain higher DHAR activity than susceptible genotypes under all the stress conditions (Figure 6iii).

The specific activity of GR was found to be upregulated in tolerant genotypes till 72 h of waterlogging stress, followed by a decrease in activity (Figure 6iv). Maximum GR activity in JLT-8 and GP-70 increased to 8.28 and 8.0 nmoles of NADP^+^ formed min^−1^ mg^−1^ of protein as compared to the control samples (3.82 and 4.25 nmoles of NADP^+^ formed min^−1^ mg^−1^ of protein) at 72 h of stress conditions, while in susceptible genotypes activity increased significantly by 48 h of waterlogging (6.06 and 5.26 nmoles of NADP^+^ formed min^−1^ mg^−1^ of protein for R-III-F6 and EC-335003, respectively), after which a drop in activity was observed till 96 h of stress. In all the stress treatments, tolerant genotypes maintained higher GR activity than susceptible genotypes (Figure 6iv).

**Figure 7:**
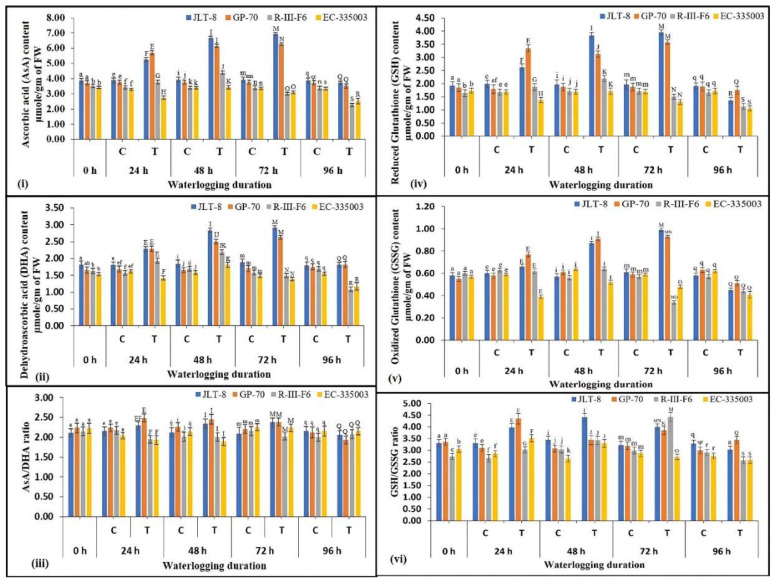
Impact of incremental waterlogging stress on the content of non-enzymatic antioxidants of the ascorbate–glutathione cycle in tolerant and susceptible sesame genotypes: (**i**) ascorbate (AsA); (**ii**) dehydroascorbate (DHA); (**iii**) ratio AsA/DHA (DHAR); (**iv**) reduced glutathione (GSH); (**v**) oxidized glutathione (GSSG); and (**vi**) ratio GSH/GSSG. Values are mean ± SD of three replicates. Different letters depict significant differences between the genotypes at *p ≤* 0.05 (Fisher LSD test).

Differential response for AsA content accumulation was found for both categories of genotypes in response to waterlogging stress. Under waterlogging, leaves of tolerant genotypes accumulated significantly higher levels of AsA than in control conditions (Figure 7i). The tolerant genotypes induced AsA content by 76.6% in JLT-8 and 66.5% in GP-70 at 72 h stress treatment, following which a sharp drop in content was observed at 96 h stressed samples. Whereas a moderate rise (29% for R-III-F6) or unchanged content (EC-335003) was noted in susceptible genotypes till 48 h of stress treatment and the content decreased significantly on higher stress treatments (Figure 7i). A similar pattern of results was also obtained for DHA content (Figure 7ii). Tolerant genotypes showed a marked increase of 55% for JLT-8, as well as GP-70, even after 72 h stress treatment, while a marginal rise of 30% and 15% was observed in susceptible genotypes at 48 h stress, in comparison to the controls, followed by a significant reduction in DHA content on exposure to further severe stress conditions (Figure 7ii). However, the leaf redox AsA/DHA ratio showed some differences (Figure 7iii). Among tolerant genotypes, a slight consistent increase in the AsA/DHA ratio was observed in JLT-8 till 72 h stress, while GP-70 showed an inconsistently higher AsA/DHA ratio till 72 h over the non-stressed samples. The AsA/DHA ratio did not show any significant differences in susceptible genotypes under stress and non-stress conditions (Figure 7iii).

GSH and GSSG levels were found to be higher by 101% and 62% in JLT-8, and 90% and 58% in GP-70, after 72 h stress treatment over the controls (Figure 7iv,v). Further severe stress reduced the contents significantly in both the genotypes. An irregular pattern was observed in the susceptible genotypes with respect to both GSH and GSSG, where the content was found unchanged at early stress conditions or decreased under subsequent conditions (Figure 7iv,v). In response to waterlogging stress, the genotypes under study exhibited a distinct regulatory pattern of the GSH/ GSSG ratio (Figure 7vi). Among the tolerant genotypes, JLT-8 showed a significant increase in GSH/GSSG in response to waterlogging till 72 h stress treatment, while a marked peak of 40% was observed in GP-70 after 24 h of waterlogging over the controls, after which the ratio decreased. However, both tolerant genotypes were able to maintain a higher ratio under all stress conditions than the control samples (Figure 7vi). Similarly, among the susceptible genotypes, R-III-F6 showed a consistent increase in GSH/GSSG ratio till 72 h stress treatment, attaining a maximum value of 4.43 (48% higher than the controls) and decreased thereafter, while a 23% increase in ratio was observed for EC-335003 in 24 h waterlogged samples, following which the ratio decreased under elevated stress conditions (Figure 7vi).

Status of Lipid peroxidation and H_2_O_2_ accumulation

**Figure 8:**
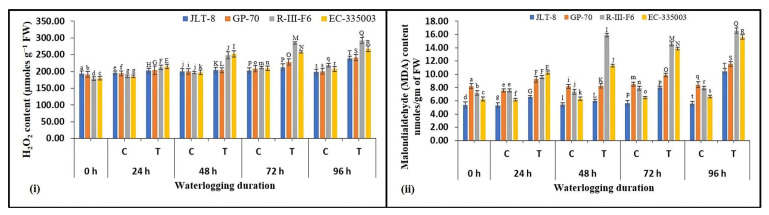
Impact of incremental waterlogging stress on the (**i**) H_2_O_2_ and (**ii**) MDA equivalent TBARS content in tolerant and susceptible sesame genotypes. Values are mean ± SD of three replicates. Different letters depict significant differences between the genotypes at *p ≤* 0.05 (Fisher LSD test).

Oxidative damage in response to waterlogging was studied in leaves of the tolerant and susceptible sesame genotypes in terms of H_2_O_2_ and MDA concentration (Figure 8i,ii). Tolerant genotypes exhibited a stable H_2_O_2_ content over a certain period of stress conditions. JLT-8 maintained H_2_O_2_ content at par with the control samples till 72 h of stress conditions, while a significant increase in content was noted at 96 h stress treatment (Figure 8i). In the case of GP-70, H_2_O_2_ content was regulated till 48 h stressed conditions, thereafter a rise in content was noted at 72 and 96 h waterlogging treated samples compared to unstressed samples, whereas a consistently increasing pattern of H_2_O_2_ content was noted in both the susceptible genotypes with increasing stress conditions (Figure 8i). The incremental waterlogging stress caused lipid peroxidation, as indicated by the increased MDA content in both tolerant and susceptible genotypes (Figure 8ii). However, it is worth noting that the increase is more significant in susceptible genotypes (peak value 109 and 135% higher in R-III-F6 and EC-335003, respectively, as compared to the controls) than tolerant genotypes (highest value was 88.5 and 37% higher than unstressed samples for JLT-8 and GP-70, respectively) after 96 h of stress treatment.

Chlorophyll Content

**Figure 9:**
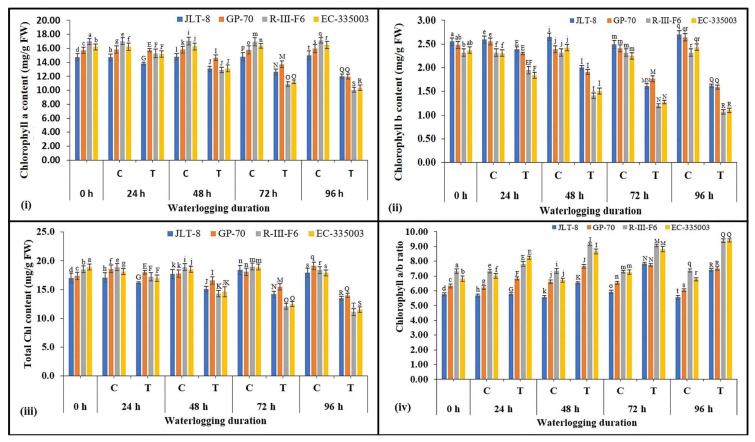
Impact of incremental waterlogging stress on the chlorophyll content in tolerant and susceptible sesame genotypes: (**i**) Chl a; (**ii**) Chl b; (**iii**) Total Chl; and (**iv**) Chl a/b ratio. Values are mean ± SD of three replicates. Different letters depict significant differences between the genotypes at *p ≤* 0.05 (Fisher LSD test).

To dissect the influence on chlorophyll contents under different times of waterlogging stress, we determined the chlorophyll a, b, total Chl content, and Chl a/b ratio of normal and after treatment for 24, 48, 72, and 96 h of waterlogging. The chlorophyll content of sesame leaves was constantly reduced with increasing duration of waterlogging in all the genotypes (Figure 9i–iv). The lowest Chl a was recorded at the highest stress treatment, i.e., 96 h, where tolerant genotypes showed 20% (JLT-8) and 25% (for GP-70) reduction, whereas susceptible genotypes exhibited a more pronounced reduction of 41% (for R-III-F6) and 37% (for EC-335003) as compared to their respective controls (Figure 9i). A similar pattern was also observed for Chl b content, where a maximum decrease of 40% in both the tolerant genotypes was recorded. However, a decrease of 54 (for R-III-F6) and 55% (for EC-335003) was observed in susceptible genotypes at the highest stress treatment compared to the controls (Figure 9ii). It can be observed that the reduction was more pronounced in Chl b content as compared to Chl a. The results also showed that the total Chl content was decreased with the continuation of waterlogging treatment in all the genotypes; however, the reduction was much more pronounced in susceptible genotypes than tolerant genotypes (Figure 9iii). Due to the comparatively higher loss of Chl b, the ratio of Chl a/b was found to be gradually larger under waterlogging conditions in both group of genotypes than their respective controls (Figure 9iv).

Proline concentration

**Figure 10:**
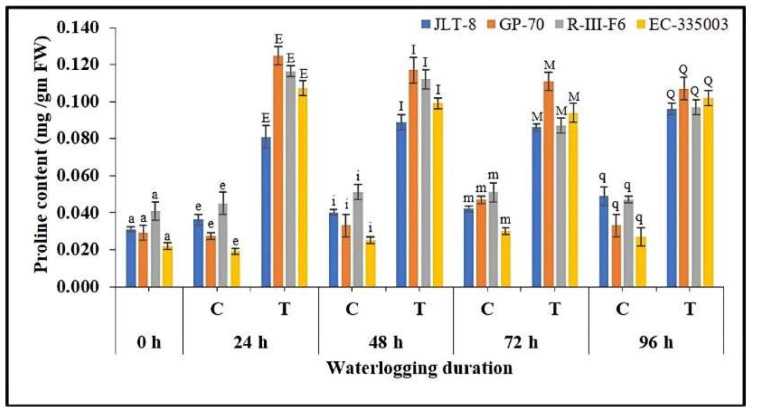
Impact of incremental waterlogging stress on the proline content in tolerant and susceptible sesame genotypes. Values are mean ± SD of three replicates. Different letters depict significant differences between the genotypes at *p ≤* 0.05 (Fisher LSD test).

The data presented in Figure 10 revealed that both the tolerant and susceptible genotypes accrued higher leaf proline content under all the stress conditions with respect to the controls. It can be observed that under initial exposure of stress conditions, i.e., in the samples assessed after 24 h waterlogging treatment, a significant increase in proline synthesis takes place in all the genotypes. An induction of 125, 363, 158, and 463% was recorded in JLT-8, GP-70, R-III-F6, and EC-335003 with respect to the control values. Subsequent higher treatments led to a decrease in proline values in all genotypes; however, the content was still significantly higher than the respective controls of each treatment (Figure 10).

Anaerobic metabolism

**Figure 11:**
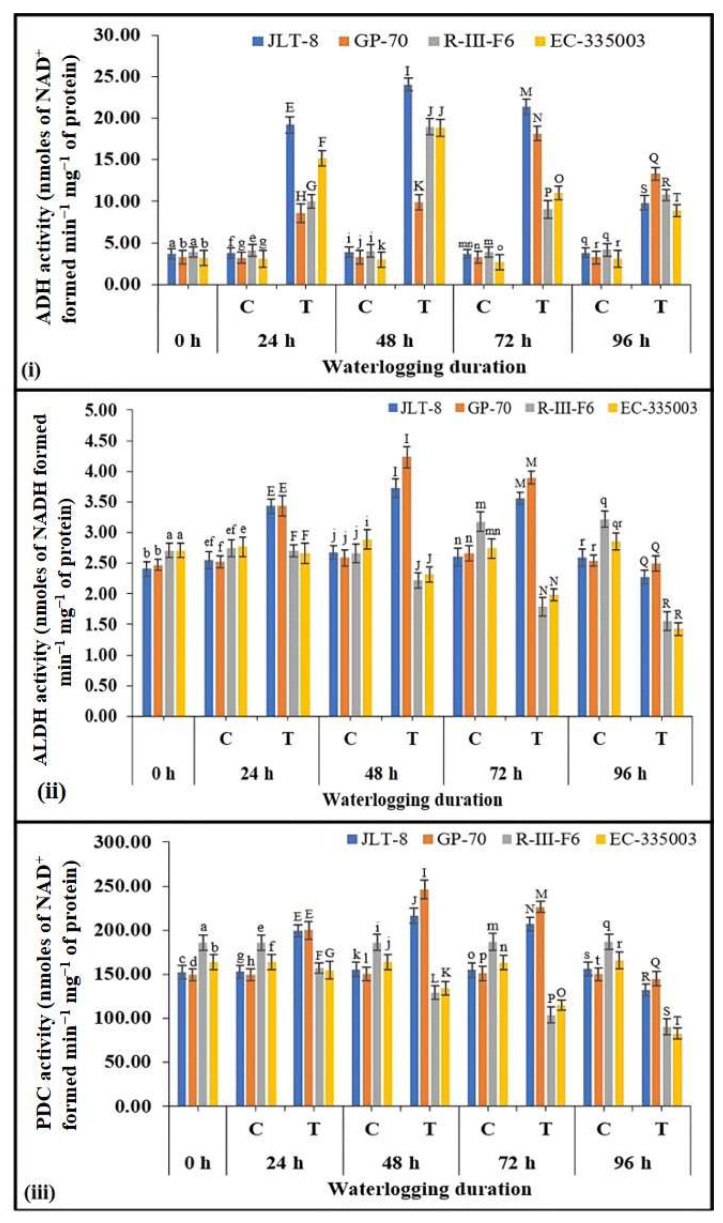
Impact of incremental waterlogging stress on the activities of anaerobic metabolism enzymes in tolerant and susceptible sesame genotypes: (**i**) alcohol dehydrogenase (ADH); (**ii**) aldehyde dehydrogenase (ALDH); and (**iii**) pyruvate decarboxylase (PDC). Values are mean ± SD of three replicates. Different letters depict significant differences between the genotypes at *p ≤* 0.05 (Fisher LSD test).

The enzyme activities involved in the anaerobic metabolism of roots revealed significant differences between the waterlogged and the control treatments of tolerant and susceptible genotypes (Figure 11i–iii). Both tolerant and susceptible genotypes showed a similar pattern for ADH activity under stress conditions (Figure 11i). In all the genotypes, a profound increase in ADH activity was observed till 48 h of stress treatment, after which activity dropped significantly, with the exception of GP-70 in which peak activity was noted at 72 h stress conditions followed by decrease in activity. JLT-8 showed a marked increase of 518%, while a 371 and 531% increase was observed for R-III-F6 and EC-335003, respectively, at 48 h stress treatment in comparison to the ADH activity of unstressed samples, whereas the ADH activity of GP-70 was highest (449% higher) at 72 h stress conditions (Figure 11i). A differential response was observed in terms of ALDH and PDC activity between tolerant and susceptible genotypes under waterlogging conditions. Tolerant genotypes exhibited an increase in ALDH and PDC activity till 48 h of waterlogging, after which the activities of both enzymes dropped with increasing stress duration. JLT-8 showed a maximum of 40% increase in ALDH activity and 39% in PDC activity at 48 h stressed conditions, whereas GP-70 showed a 64% increase in ALDH activity and 63.5% increase in PDC activity at same stage compared to their respective controls (Figure 11ii,iii). In contrast, the activity of both enzymes was found to be decreasing in both susceptible genotypes under all the stress conditions. The activities of ALDH and PDC were found to be decreased by 52% in R-III-F6 and 50% in EC-335003 at highest waterlogging treatment (i.e., 96 h) with respect to their activity at unstressed conditions (Figure 11ii,iii). Under the conditions of waterlogging, tolerant genotypes were able to maintain higher ALDH and PDC activity than susceptible genotypes.

### 2.3. Principal Component Analysis

**Figure 12:**
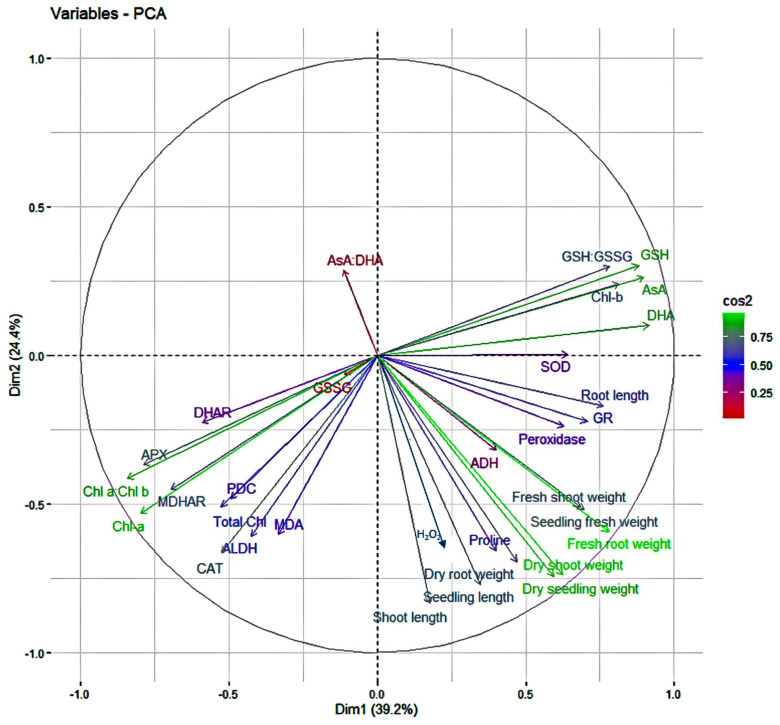
Biplot of different characters of sesame genotypes under control conditions.

**Figure 13:**
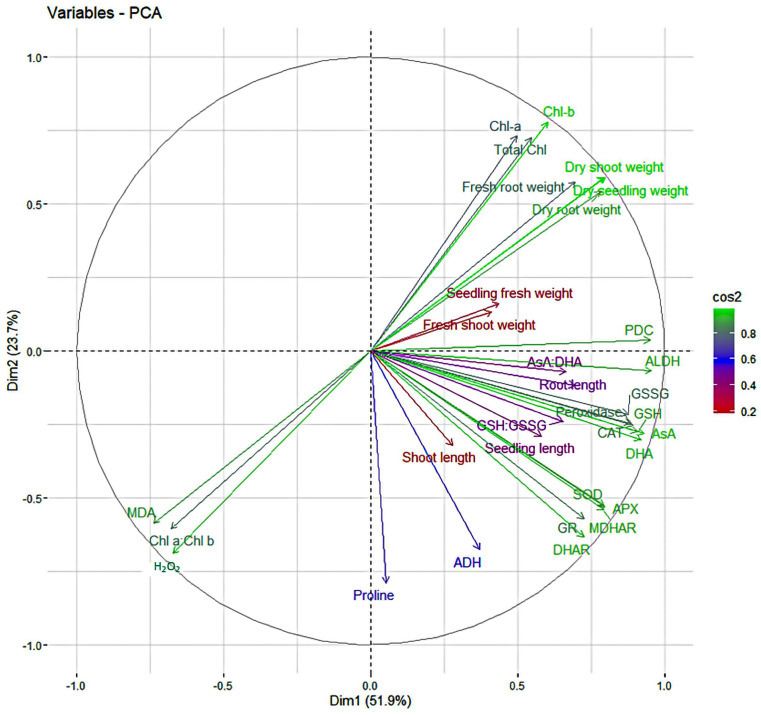
Biplot of different characters of sesame genotypes under waterlogging stress conditions.

The principal component analysis (PCA) was performed based on different physiological and biochemical traits of the sesame genotypes in optimal and stress environments. Principal component analysis revealed that the first five PCs had an eigen value of more than one, which contributed 90.38 percent variability in optimal condition for different traits, whereas for waterlogging stress conditions, 88.44 percent variability is due to first four PCs, which had an eigen value of more than one (Table 1). The overall variation for genotypes in optimal condition had a contribution of 39.21 percent from PC1 and 24.41 percent from PC2, whereas in stress conditions PC1 describes 51.94 percent and PC2 elucidates 23.67 of the overall variation. There are two major antioxidants like H_2_O_2_ and MDA, which are the markers for oxidative stress such as waterlogging stress. The principal component biplot for the optimal condition (Figure 12) showed that H_2_O_2_ had negative correlation with AsA:DHA, GSH:GSSG, and GSH; and positive correlation with MDHAR, Chl a, PDC, Total Chlorophyll, ALDH, CAT, MDA, proline, ADH, peroxidase, GR, SOD, shoot length, seedling length, dry root weight, dry seedling weight, dry shoot weight, fresh root weight, seedling fresh weight, root length and fresh shoot weight, DHAR, APX, Chl a:Chl b, GSSG, AsA and Chl*b*. MDA had negative correlation with AsA:DHA, GSH:GSSG, GSH, AsA, Chl-b, DHA, SOD, GR, and root length; and positive correlation with DHAR, GSSG, APX, Chl a:Chl b, Chl a, MDHAR, PDC, Total Chl, CAT, ALDH, H_2_O_2_, Proline, ADH, peroxidase, shoot length, seedling length, dry root weight, dry seedling weight, dry shoot weight, fresh root weight, seedling fresh weight and fresh shoot weight. However, in waterlogging stress conditions (Figure 13) H_2_O_2_, MDA and Chl a:Chl b showed negative correlation with Chl a, Chl b, total chlorophyll, PDC, ALDH, AsA:DHA, GSSG, GSH, peroxidase, AsA, CAT, DHA and GSH:GSSG, dry shoot weight, fresh root weight, dry seedling weight, dry root weight, seedling fresh weight, fresh shoot weight, seedling length, and root length; and positive correlation with proline, ADH, DHAR, MDHAR, GR, APX, and SOD.

## 3. Discussion

Waterlogging is one of the most significant environmental stresses, causing biochemical, physiological, morphological, and anatomical changes that result in severe agricultural production losses [26]. Climate change is anticipated to cause long periods of minimal rainfall, followed by strong and protracted precipitation, resulting in severe floods [27]. It is therefore imperative to develop genotypes that are flood-tolerant in order to circumvent these challenges. Despite the fact that sesame is extremely sensitive to waterlogging, there are significant variances between genotypes [21,28]. As a result, in order to evaluate the flooding tolerance of various sesame genetic resources, they must be screened under varying waterlogging conditions. Although much work has addressed the mechanisms of waterlogging tolerance in the crops, like rice [29], *Arabidopsis* [30], and maize [5,12], only a few studies are reported regarding hypoxic adaptation and tolerance in sesame genotypes at particularly prone vegetative stage taking antioxidant system and anaerobic metabolism) together in consideration. In the present study, a large and diverse pool of sesame germplasm was screened for their waterlogging tolerance at a stage which frequently encounters waterlogging stress. The duration of the treatments was also carefully selected, keeping in view the period of stress a crop generally faces under natural conditions. Among the screened genotypes, four genotypes of highly contrasting response against waterlogging stress, i.e., highly tolerant and highly susceptible, were subjected to incremental waterlogging stress treatments for the understanding of the biochemical basis of the mechanism for their respective behavior under waterlogging conditions. Authors have taken into account particularly two major biochemical systems, viz., antioxidant and anaerobic metabolism, along with some other relevant characters such as photosynthetic ability, osmotic regulation, and some key morphological characters and adaptation parameters in order to gain insight about the mechanism of tolerance against waterlogging stress. A comprehensive analysis of the genotypes under systematic stress treatments has revealed significant variation in responses in terms of biochemical adjustments for tolerant and susceptible genotypes, which might have played a pivotal role in the survival of the plants under severe stress conditions.

The conditions of soil waterlogging exerted detrimental effects on seedling growth in both tolerant as well as susceptible genotypes. Various studies on different crop species have corroborated these findings, indicating that prolonged waterlogging during the seedling stage results in the decreased length of both root and shoot, as well as reduced fresh and dry mass of both root and shoot tissues (wheat [31,32], chickpea and faba bean [33], and maize [34]). Hence, the longer the waterlogging duration, the greater the reduction in seedling growth noted in both group of genotypes. In a recent study conducted by Kyu et al. [35], it was observed that temporary waterlogging resulted in a reduction of soil redox potential. This phenomenon led to a delay or inhibition of seed germination, hindered the establishment of seedlings, and adversely affected the growth of both shoots and roots. WTC of fresh and dry seedling weight, calculated on the basis of seedling length, revealed that tolerant genotypes exhibited higher WTC and indicated better adaptation and endurance of tolerant genotypes under waterlogging conditions. Extended periods of inundation have been demonstrated to hinder the developmental progress of seedlings by the fourth day. Specifically, plant varieties lacking resistance to hypoxia exhibit sluggish growth or cease growth entirely under such conditions, as documented by Wu et al. [36]. In accordance with the studies conducted by Habibullah et al. [37] and Olorunwa et al. [38], it has been established that Waterlogging Tolerance Coefficients (WTCs) and seedling traits serve as precise indicators for the preliminary evaluation of different genotypes’ resilience and vulnerability to flood conditions. Elevated numerical values are indicative of enhanced tolerance to waterlogged environments when contrasted with lower values. The research by Habibullah et al. [37] utilized WTCs to assess sixteen sesame genotypes, discerning their levels of tolerance and susceptibility to waterlogging during the seedling phase. Our findings align with the research conducted by Kaur et al. [6], wherein they similarly documented significant modifications in root morphology. Specifically, the susceptible maize inbred line I110 exhibited diminished root length and biomass under waterlogged conditions compared to the tolerant line, I172. Various studies conducted on different crops have revealed that genotypes exhibiting tolerance tend to experience reduced damage in terms of biomass during waterlogging conditions [38,39,40].

One of the predominant and vital adaptive characteristics enabling plants to endure waterlogged soil environments is the formation of adventitious roots. These morphological adaptations facilitate the absorption of oxygen by submerged tissues, mitigate hypoxic circumstances, and enhance plant viability in frequently waterlogged soils [41]. The development of adventitious roots in both groups of genotypes indicates the morphological adjustment of tolerant as well as susceptible genotypes in order to cope up with a regular supply of oxygen to sustain the stress period. However, a more rapid and vigorous development of aerial roots in tolerant genotypes indicates its ability to adapt faster and better than the susceptible genotype, and hence may be responsible for its survival. In the study conducted by Wei et al. [23], analogous results were documented, demonstrating the occurrence of earlier and more robust development of adventitious roots in the waterlogging-tolerant sesame accession ZZM2541 in comparison to its waterlogging-susceptible counterpart, Ezhi-2. Wang et al. [21] observed comparable results wherein flooding-tolerant varieties, Wild No. 7 and Yuzhi No. 1, exhibited a significant increase, approximately four to five times, in the formation of adventitious roots in comparison to less flooding-tolerant genotypes, Danbackaggce and Suiping Xiaozhihuang. Notably, the latter genotypes displayed a minimal increment in the number of adventitious roots under similar conditions. In the study conducted by Islam and Khatoon [42], the emergence of adventitious roots in four sesame genotypes (Rajshahi Khoyeri, KistotilChapai, KathtilChapai, and Binatil-2) after 72 h of waterlogging was observed. This phenomenon was linked to the genotypes’ survival mechanism in hypoxic conditions. The present research findings align with a prior study by Kim et al. [43], wherein both tolerant (PI408105A) and susceptible (S99-2281) soybean genotypes exhibited adventitious root formation under waterlogged conditions. Notably, the development of adventitious roots was more pronounced in tolerant genotypes compared to their susceptible counterparts, corroborating the results obtained in the current study.

From the results, it is evident that however the genotypes induced their antioxidant defense mechanism to cope up the oxidative stress, the tolerant genotypes were able to maintain more pronounced and prolonged antioxidant response and more efficiently mitigate the oxidative stress injury in their cells. The coordinated action of SOD, POX, and CAT must have played an instrumental role in providing tolerance to the tolerant sesame genotypes even under severe stress conditions. The SOD is a principal ROS scavenging enzyme which catalyzes the dismutation of O_2_^•−^ to H_2_O_2_ and O_2_. The increased SOD activity has been correlated with increased protection from the oxidative damage in barley under drought stress conditions [44]. The ability of tolerant sesame genotypes to scavenge superoxide anions more efficiently under waterlogged conditions owing to its better antioxidant system, as evident by the higher activities of SOD, POX, and CAD, might act as an indicator. It has been proposed that the increased expression of genes involved in the scavenging of reactive oxygen species, such as SOD and APX, contributes to an enhanced antioxidative defense mechanism. This augmentation ultimately leads to higher tolerance against abiotic stressors, as evidenced by Lee et al. [45]. Numerous research studies have emphasized the importance of strengthening the antioxidant defense system, which includes antioxidant enzymes and metabolites, combating oxidative stress; notably, key detoxifying enzymes such as SOD, POX, and CAT work in collaboration with APX and GR within the ascorbate–glutathione cycle, aiding in the elimination of ROS during abiotic stress episodes [5,9,34,46,47,48].

Habibullah et al. [37] also observed that the enzymatic antioxidants SOD, POX, and CAT exhibited elevated activity levels in waterlogging-tolerant sesame genotypes (specifically BD-7008 and BD-6985) when compared to their sensitive counterparts (BD-6996 and JP 01811). This increase in enzymatic antioxidant activities indicated a more efficient balance between the ROS produced due to waterlogging and the detoxification processes. Consequently, this enhanced equilibrium empowered the plants to better endure the stressful conditions induced by flooding. Our results also align with the research conducted by Wei et al. [23], who established that the waterlogging-resistant sesame variant ZZM2541 effectively sustains a superior equilibrium between the generation of ROS and their detoxification. This equilibrium is attributed to the heightened levels of SOD and CAT observed in ZZM2541 in comparison to the waterlogging-susceptible sesame accession Ezhi-2. The study also revealed rapid accumulation of CAT activity during the initial phases of waterlogging stress in ZZM2541, maintaining elevated levels throughout the later stages. This suggests that the scavenging of H_2_O_2_ by CAT serves as a crucial mechanism employed by ZZM2541 to mitigate waterlogged stress effectively. Our findings are also consistent with previous findings of Anee et al. [3], Wang et al. [49], Keya et al. [50], Sairam et al. [51], Damanik et al. [52], Zhu et al. [53] and Xu et al. [54], since they reported comparatively higher enzymatic antioxidant activities in the waterlogging-tolerant genotypes than the sensitive ones.

The Foyer–Halliwell–Asada pathway, also recognized as the ascorbate–glutathione pathway, constitutes an essential component of the ROS homeostasis mechanism in plants, specifically designed for the detoxification of H_2_O_2_. This pathway includes pivotal antioxidant metabolites such as ascorbate, glutathione, and NADPH, in conjunction with the enzymes that facilitate their interactions. Within this intricate system, plants deploy a diverse array of non-enzymatic scavengers. These scavengers collaborate with antioxidant enzymes to effectively counteract the detrimental impacts of ROS and mitigate cellular damage, particularly under circumstances of oxidative stress. As an integral element of the ascorbate–glutathione (AsA–GSH) cycle for H_2_O_2_ detoxification, ascorbate serves as the substrate for APX, a prominent non-enzymatic antioxidant enzyme [55]. Enzymes such as APX, MDHAR, DHAR, and GR play pivotal roles within the AsA-GSH cycle, a vital biochemical pathway that effectively utilizes AsA and GSH to neutralize ROS. These enzymes actively eliminate ROS and, in turn, regenerate AsA and GSH for continued detoxification. Specifically, MDHAR is essential for the regeneration of AsA and the maintenance of a reduced biochemical pool, ensuring its availability for ROS neutralization. Concurrently, GR orchestrates the management of the GSH pool, enhancing the elimination of H_2_O_2_ during stress-induced conditions.

All the components of the system were analyzed in the tolerant and susceptible genotypes. The results clearly indicated that both groups of genotypes induce all the enzymes (APX, MDHAR, DHAR, and GR) involved in ascorbate–glutathione based H_2_O_2_ detoxification. Where susceptible genotypes showed a moderate upregulation in activities till 48 h of stress conditions, following which activities dropped significantly, the tolerant genotypes showed a much-pronounced rise in activities and maintained higher enzyme levels even after 72 h and 96 h of waterlogging stress compared to the controls. A similar pattern was also observed for AsA and DHA content; the AsA/DHA ratio was increased only in tolerant genotypes while susceptible genotypes did not show any significant change. GSSG and GSH were also found to be increased only in tolerant genotypes up to some extent, while either no change or a decrease in content was noted for susceptible genotypes. The GSH/GSSG ratio showed some variable pattern however, with the tolerant genotypes exhibiting a higher ratio than the controls. Wei et al. [23] observed a substantial 5.7-fold rise in the activity of APX in the waterlogging-tolerant sesame accession ZZM2541. In contrast, the waterlogging-susceptible accession Ezhi-2 exhibited only a modest 1.5-fold increase in APX activity after 48 h of waterlogging stress. The researchers posited that the swift and enduring buildup of APX in the tolerant accession plays a pivotal role in effective H_2_O_2_ scavenging by APX, thereby ensuring the plant’s survival under waterlogging conditions. An increase in APX and MDHAR activity has been reported in sesame plants under increasing duration of waterlogging stress [3]. In surviving barley cultivars subjected to drought stress, it was observed that heightened activity levels of enzymes such as APX, GR, and DHAR were associated with a reduced accumulation of H_2_O_2_ [44]. Likewise, higher GR activity under chilling and hypoxic stress has also been reported in tolerant maize genotypes in comparison to susceptible genotypes [56]. GR plays a crucial role in the regulation of oxidative stress by facilitating the transformation of oxidized glutathione (GSSG) into its reduced form (GSH) and upholding an elevated GSH/GSSG ratio [57]. In the study conducted by Hossain et al. [58], it was observed that citrus plants, specifically citrumelo CPB4475, exhibited an enhanced antioxidant response when subjected to oxidative stress caused by flooding. The coordinated antioxidant activity involved the simultaneous increase in SOD and CAT activities, along with a modulation of the ascorbate–glutathione cycle. This mechanism enabled the plants to manage oxidative stress up to a certain threshold. The researchers concluded that while higher APX activity or discrete elevations in AsA or glutathione concentrations were present, they appeared to be ineffective in maintaining consistently low levels of oxidative damage.

Several other reports also confirmed the findings of the current study [59,60,61,62]. The glutathione levels were observed to exhibit a notable increase in plants subjected to prolonged waterlogging stress lasting four days or more [3]. Elevated activities of MDHAR and DHAR enzymes under stress conditions lead to augmented AsA levels, while increased activity of GR enhances the GSH/GSSG ratio [63]. GSH functions by inhibiting the generation of peroxides and free radicals via its unbound thiol group [64]. Indeed, the AsA/DHA ratio strongly increased in the tolerant sesame genotypes, demonstrating improved redox homeostasis in these genotypes. A second redox buffer, i.e., the ratio of GSH/GSSG, also increased significantly in the tolerant genotypes, maintaining the redox status in these genotypes under waterlogged conditions. Abid et al. [65] observed a higher ratio of GSH in wheat cultivars displaying tolerance in comparison to their sensitive counterparts. Previous research has highlighted the coordinated regulation of two enzymes, MDHAR and DHAR. MDHAR assumes a significant role under stressful conditions, whereas DHAR’s functionality becomes prominent only in situations marked by decreased ascorbate synthesis [66] or an elevated presence of dehydroascorbate [67]. DHAR’s enzymatic activity necessitates increased GR activity for GSH production; otherwise, it leads to the accumulation of GSSG, which serves as an oxidative marker [68]. A higher level of enzymes in tolerant genotypes, along with non-enzymatic metabolites and their ratio, very well explains its better response; hence, plant survival under increasing duration of waterlogging stress.

The findings outlined above were further corroborated and validated by the examination of oxidative stress markers, H_2_O_2_ and MDA levels. When subjected to waterlogging stress, tolerant genotypes exhibited controlled H_2_O_2_ levels, indicating a reduced extent of oxidative damage. The nearly stable or slightly elevated levels of H_2_O_2_ and MDA in waterlogging-tolerant sesame genotypes under prolonged waterlogging conditions can be attributed to elevated activities of SOD, POX, and CAT, and a highly efficient ascorbate–glutathione pathway within their tissues. In contrast, susceptible genotypes exhibited a moderate rise in enzyme activity within a limited time period. It can be inferred that in tolerant genotypes, the detoxification of H_2_O_2_ was effectively regulated through the coordinated action of various components of the antioxidant defense system. This phenomenon likely accounts for the heightened sensitivity observed in genotypes R-III-F6 and EC-335003 when exposed to waterlogged environments. Additionally, the measurement of MDA content in plant tissues serves as a widely accepted indicator of lipid peroxidation induced by diverse abiotic stresses. The sustained level of lipid peroxidation signifies a plant’s resilience against environmental challenges such as waterlogging. Similar results pertaining to lipid peroxidation and the antioxidative system function have been reported by other researchers [3,23,37,49,50].

To assess the photosynthetic efficiency of contrasting genotypes under waterlogged conditions, the changes in chlorophyll content with increasing stress duration was also determined. It was found that the pigment level was at par with the controls in the case of tolerant genotypes, while a reduction in the same was noted for susceptible genotypes, which may, consequently, have affected their photosynthesis ability under waterlogging conditions. Chlorophyll facilitates the process of photosynthesis by capturing the light energy [69]. The process of leaf senescence is initiated as a response to waterlogging-induced stress, leading to a decrease in chlorophyll content [70]. In the context of waterlogging-induced stress, the discoloration of leaves may manifest as a consequence of diminished leaf nitrogen content, compromised nodulation, and reduced nitrogen fixation [71]. The ability to withstand waterlogging stress can be assessed through enhanced chlorophyll retention. Therefore, genetic diversity in chlorophyll concentration proves advantageous in identifying tolerant genotypes under waterlogged conditions. Previous studies on different sesame genotypes have also reported consistent results under gradually increasing waterlogging stress, confirming the significance of chlorophyll variation in stress resistance [3,23,37,72].

Numerous plant species exhibit the capacity to synthesize a variety of compatible osmolytes in response to a spectrum of abiotic stress conditions, encompassing substances like proline and various sugars. These osmolytes serve as osmoprotectants, enhancing the plant’s ability to withstand environmental stress factors [73]. In the present study, tolerant as well as susceptible genotypes accumulated a significantly higher content of proline in their leaf tissues under all stress regimes. In the context of stress response, proline serves a protective role by directly neutralizing ROS, stabilizing proteins and antioxidant enzymes, maintaining the balance of intra-cellular redox homeostasis (specifically the ratio of NADP^+^/NADPH and GSH/GSSG), and enhancing cell signaling while safeguarding photosynthetic pigments [74,75]. Several researchers have reported that the increased accumulation of proline in response to waterlogging stress is regarded as a significant biochemical adaptation that serves to mitigate the deleterious consequences of waterlogging, ultimately leading to enhanced plant performance [9,12,37,50,76,77].

In conditions of oxygen deprivation, the process of ATP synthesis via oxidative phosphorylation is impeded, necessitating the production of ATP through fermentation. Plants exhibiting higher tolerance to waterlogging have been observed to exhibit a more effective pathway of alcoholic fermentation [78]. Therefore, as a part of the present study, activities of three vital anaerobic metabolism enzymes, i.e., ALDH, PDC, and ADH, were investigated and exhibited a noticeable variation between the waterlogging tolerant and susceptible sesame genotypes. Where tolerant genotypes exhibited induced activities of all three enzymes, upregulation of only ADH was observed in susceptible genotypes. Ethanol is the main end product of anaerobic metabolism in plants. Elevated activity of ADH might be essential to deter the accumulation of acetaldehyde, a substance with potential toxicity [79]. In the context of alcoholic fermentation, ADH plays a pivotal role in the regeneration of NAD^+^, crucial for sustaining glycolysis. In pigeonpea plants, it has been observed that the correlation between the survival rate and the production of acetaldehyde during submergence is notably unfavorable [80]. Studies investigating *adh1* mutations in Arabidopsis and maize have elucidated the pivotal role of ADH activity in providing short-term resistance against waterlogging conditions [81]. Ethanol accumulation was observed to be re-metabolized in anaerobic environments through the actions of ALDH and acetyl-CoA synthase, leading to the formation of acetaldehyde, which subsequently undergoes breakdown into acetate and acetyl-CoA [82]. Based on reported findings, the excessive amplification of the stress-induced acetaldehyde dehydrogenase gene (*Ath-ALDH3*) effectively constrains the augmentation of acetaldehyde within plant organisms, functioning as an innovative detoxification mechanism [83]. The PDC enzyme facilitates the decomposition of pyruvate into carbon dioxide and acetaldehyde, which serves as a precursor for ethanol synthesis. This enzymatic process represents a pivotal juncture in anaerobic metabolism. PDC has been recognized as a decisive factor limiting the rate of ethanol generation from pyruvate in oxygen-deprived environments [84]. A high level of ADH and PDC enzymes may be essential to impede the accumulation of acetaldehyde, a substance with potential toxicity [85].

Elevated PDC activity leads to intensified carbon flux within the ethanol pathway, augmenting the sizes of acetaldehyde and ethanol pools. Consequently, there is a subsequent rise in ATP and NAD^+^ production. In contrast to lactic acid fermentation, which induces cytoplasmic acidosis, the persistent synthesis of ATP through ethanolic fermentation confers advantageous outcomes. In a recent investigation, researchers have documented an elevated enzymatic activity of ADH and PDC in kiwifruit (*Actinidia* spp.) rootstocks known for their waterlogging tolerance when subjected to waterlogging-induced stress conditions. This aggravated enzymatic activity corresponds to a reduced accumulation of lactic acid and ethanol within the plant tissues [86]. Furthermore, it has been previously demonstrated that the upregulation of ADH or PDC enzymes can enhance resistance to low-oxygen stress in Arabidopsis [87]. Expression analysis studies have elucidated heightened transcriptional activity of genes participating in alcoholic fermentation pathways, notably *pyruvate decarboxylase 1 [Pdc1]*, *Pdc2* and *adh1*, as well as genes associated with lactic acid fermentation (*ldh1*), in response to a reduction in external oxygen concentration to 5% [88].

Goyal et al. [89] observed a substantial elevation in the enzymatic activities of PDC, ADH, and ALDH in the hypoxic roots of maize genotypes I 167 (flooding tolerant) in comparison to LM 5 (flooding susceptible). This finding underscores the superior anaerobic tolerance mechanism exhibited by the flooding-tolerant genotype I 167, highlighting its efficiency in adapting to oxygen-deprived conditions. In a recent study conducted by Gao et al. [90], it was revealed that the waterlogging tolerance observed in the wild *A. valvata* germplasm is linked to elevated levels of PDC and ADH, along with enhanced antioxidant capacity. This finding suggests a pre-adaptive mechanism enabling sustained energy generation amidst subsequent periods of anoxia. Based on the findings, it is evident that elevated levels of ADH, ALDH, and PDC enzymes in the roots of tolerant genotypes potentially facilitate sesame survival under adverse conditions. This enhancement in enzyme activities enables the utilization of fermentation pathways, converting carbohydrates into energy even in the absence of oxygen, thus ensuring the production of ATP. Furthermore, this mechanism likely serves to mitigate the harmful effects caused by the accumulation of toxic acetaldehyde during waterlogged conditions. Consequently, the augmented enzymatic activities contribute to sustaining ATP synthesis in oxygen-deprived environments, enhancing the overall survival strategy of sesame plants.

Plants mitigate various abiotic stresses by maintaining the level and combination of different morphological, physiological, and biochemical traits. To know which trait or combination of traits would mitigate stress better, it is necessary to find a group of principal traits which can be obtained by principal component analysis (PCA) [91]. PCA showed that PC1 and PC2 are the important principal components as they contributed to the maximum variance in both optimal (63.62%) and stress conditions (75.62%), with an eigen value of more than one, which implies that the traits have a narrow angle in PC1 and PC2, and which may be used for indirect selection of the genotypes to mitigate the stress by creating better genetic recombinants [3,92,93]. However, most of the traits which are necessary for the survival of seedlings were showing positive correlation with H_2_O_2_ and MDA in optimal condition, and had shown negative correlation in stress conditions. It indicates that they may play a very important role in mitigating the prevailing stress by balancing their level in the cell. Similar results were observed by Sharma et al. [94], Anee et al. [3], Hasanuzzaman and Fujita [95], Nakano and Asada [55], Hasanuzzaman et al. [96], Lin et al. [97], and May et al. [98] in sesame and other crops for different stresses.

## 4. Materials and Methods

### 4.1. Screening of the Plant Material

The 142 lines of sesame (including cultivated varieties, landraces, advanced breeding lines, and other elite germplasm) (Table 1) were provided by the Department of Genetics and Plant Breeding, Banda University of Agriculture and Technology. The seeds were surface-sterilized using a 0.1% mercuric chloride solution for 5 min and then rinsed with distilled water before being used. The experiment took place in a regulated environment at a temperature of 30 ± 2 °C with 12 h of darkness and 12 h of continuous illumination at a photosynthetically active radiation (PAR) of 200 µmol m^−2^ s^−1^. For screening of the germplasm, the cup method was employed, which had been previously standardized for evaluating waterlogging stress in maize [99]. Sesame seedlings were grown in reusable plastic cups (250 cm^3^) with three 5.0 mm diameter perforations at the base. These cups were filled with a mixture of farmyard manure (FYM) and siphoned soil in a 1:1 ratio (volume/volume) up to 220 cm^3^, and then placed in plastic trays measuring 50 × 30 × 10 cm. Fertilizer containing nitrogen, phosphorus, and potassium (NPK) was applied as a basal dose and thoroughly mixed into the soil, the amount of which was calculated based on the soil weight. Each cup’s weight was measured after filling to ensure consistent soil quantity and constant moisture. Seedlings in the early vegetative stage were chosen for their uniformity and then exposed to waterlogging. The experiment followed a randomized split-plot design with three replications. Following fifteen days of regular growth, when the second leaf was fully expanded, five uniform seedlings were waterlogged by placing cups in a tray filled with water, ensuring the soil surface remained at least 30 mm below water throughout the stress period. As a control group, a set of genotypes was kept at normal moisture levels. The water level in the cups was consistently upheld through frequent replenishment for a period of seven days. The onset of waterlogging stress-induced symptoms, such as leaf yellowing (chlorosis), leaf browning, drooping, wilting, and leaf necrosis, was used as criteria for the screening of the genotypes. This screening experiment was repeated thrice, and on the basis of the manifestation of these symptoms and the survival of the seedlings under waterlogged conditions, the genotypes were grouped as tolerant, moderately tolerant, susceptible, and highly susceptible (Table 2).

Based on the above results, a subset of four contrasting genotypes, i.e., two highly tolerant (JLT-8, and GP-70) and two highly susceptible (R-III-F6 and EC-335003), were selected for further incremental waterlogging stress treatment study. The plants were grown in similar conditions and 15 day old seedlings were selected for uniformity and then waterlogging treatment. With three replications, the experiment was conducted as a fully randomized split-plot. The five waterlogging treatments (0, 24, 48, 72 and 96 h of waterlogging) and the four genotypes were used as a sub-plot and main-plot, respectively. The waterlogging treatment was given as mentioned above. Throughout the experiment, optimal moisture levels were maintained for the control group of plants. The leaf and root samples were used for the estimation of various biochemical parameters under study.

### 4.2. Seedling Measurements

Various morphological parameters, such as root and shoot length, seedling length (cm), and fresh and dry weight (g) of roots, shoots and complete seedlings, were measured for each genotype under the control and waterlogged conditions after the end of treatments. Observations related to seedling characteristics were meticulously documented for five plants per experimental repetition, with each treatment being replicated thrice. Furthermore, the waterlogging tolerance coefficient (WTC) for each genotype was quantified as a percentage employing the following formula:

$$WTC\;\left(weight\right)=\frac{\mathrm{Seedling}\;\mathrm{fresh}/\mathrm{dry}\;\mathrm{weight}\;\mathrm{under}\;\mathrm{waterlogging}\;\mathrm{conditions}}{\mathrm{Seedling}\;\mathrm{fresh}/\mathrm{dry}\;\mathrm{weight}\;\mathrm{under}\;\mathrm{control}\;\mathrm{conditions}}\times 100\;$$

$$WTC\;\left(length\right)=\frac{\mathrm{Seedling}\;\mathrm{length}\;\mathrm{under}\;\mathrm{waterlogging}\;\mathrm{conditions}}{\mathrm{Seedling}\;\mathrm{length}\;\mathrm{under}\;\mathrm{control}\;\mathrm{conditions}}\times 100$$

### 4.3. Extraction and Estimation of Enzymes

#### 4.3.1. Extraction and Estimation of Antioxidant Enzymes

The extractions were performed in triplicate at 4 °C and subsequently analyzed in duplicate at 37 °C. SOD, POX, and GR enzymes were extracted through homogenization of samples in 0.1 M phosphate buffer (pH 7.5) and supplemented with 1 mM ethylenediaminetetraacetic acid (EDTA), 1% polyvinylpyrrolidone (PVP), and 10 mM β-mercaptoethanol. CAT and APX were extracted using 0.05 M phosphate buffer (pH 7.5) with 1% PVP, as described by Kaur et al. [46]. Subsequently, the homogenates were subjected to centrifugation at 10,000× *g* for 20 min, and the resulting supernatant was employed for subsequent assays.

The SOD assay system utilized a solution comprising 100 mM Tris-HCl buffer (pH 8.2), 6 mM EDTA, and a 6 mM pyrogallol solution, as reported by Marklund and Marklund in 1974 [100]. This method was subjected to specific alterations in the concentrations of these components, according to the protocol outlined by Singh et al. [101]. The absorbance shift at 420 nm was monitored at 30-s intervals over a duration of 3 min. One unit of enzyme activity was defined as the quantity of enzyme necessary to induce 50% auto-oxidation of pyrogallol observed in a blank sample.

The GR assay mix involved the addition of a 200 mM potassium phosphate buffer (pH 7.5) containing 0.2 mM EDTA, 1.5 mM MgCl_2_ (1.5 mM), and 0.2 mL NADPH (0.025 mM) to the enzyme extract, after which 0.25 mM of oxidized glutathione was added in a quartz cuvette [102]. Modifications were made to the original procedure, including the substitution of the phosphate buffer (0.2 M, pH 7.5) for Tris-HCl (0.1 M, pH 7.4), and adjustments to the quantities of other assay components [103]. Decreases in absorbance at 340 nm were recorded at 30-sec intervals for up to 3 min. The enzymatic activity of GR was quantified as units, where one unit represented the formation of nmoles of NADP^+^ min^−1^ g^−1^ of fresh weight (FW). The calculation involved using an extinction coefficient (€_NADPH_) of 6.22 mM^−1^ cm^−1^.

The POX assay solution comprised 0.05M guaiacol dissolved in 100 mM phosphate buffer at pH 6.5, to which enzyme extract was added, followed by the introduction of 0.8 M H_2_O_2_, as outlined by Shannon et al. [104]. As a blank, the reaction mixture devoid of H_2_O_2_ was used. The reaction was started with the addition of H_2_O_2_, and a rate of absorbance shift at 470 nm was observed for a duration of 3 min at 30-sec intervals. POX activity was quantified as the alteration in absorbance/min/g of fresh weight (min^−1^ g^−1^ of FW).

The APX assay mixture comprised a 50 mM sodium phosphate buffer (pH 7.0) solution containing 0.5 mM ascorbic acid and enzyme extract, as described by Nakano and Asada [105]. The absorbance measurements were conducted at 290 nm using a spectrophotometer at 30-sec intervals for a duration of 3 min. The extinction coefficient of monodehydroascorbic acid (MDAA) was determined to be 2.8 mM^−1^cm^−1^. APX activity was quantified in terms of nmoles of MDAA formed min^−1^ g^−1^ of FW.

The CAT activity was assessed by preparing a reaction mixture comprising 50 mM sodium phosphate buffer of pH 7.5, to which enzyme extract was added. The reaction was initiated by introducing 1 mL of H_2_O_2_, and the consumption of H_2_O_2_ was monitored at 30-sec intervals over a duration of 3 min. This monitoring was conducted by measuring the reduction in absorbance at 240 nm, as suggested by Chance and Maehly [106]. The extinction coefficient for H_2_O_2_ was determined to be 0.0394 mM^−1^cm^−1^. CAT activity was quantified as μmoles of H_2_O_2_ decomposed per minute per gram of FW.

In the MDHAR and DHAR assays, tissue samples weighing between 0.25 to 0.5 g were subjected to extraction using 100 mM potassium phosphate buffer (pH 7.5) supplemented with 2% PVP, 1 mM EDTA, and 1 mM ascorbate, the latter added immediately prior to utilization. In the MDHAR assay, the reaction mixture comprised 50 mM potassium phosphate buffer (pH 7.5), 2 mM MgCl_2_, 10 mM NaCl, 0.1 mM NADH, 400 mM sucrose, 2.5 mM ascorbate, and 0.25 units of ascorbate oxidase. The disappearance of NADH was monitored at 340 nm and quantified using the molar extinction coefficient for NADH, which is 6.22 mM^−1^ cm^−1^. For the DHAR assay, the reaction mixture consisted of 50 mM potassium phosphate buffer (pH 7.0), 0.1 mM EDTA, 2.5 mM reduced glutathione (GSH), and 0.2 mM dehydroascorbate. The formation of ascorbate was measured at 265 nm and quantified using the molar extinction coefficient for ascorbate, which is 14 mM^−1^ cm^−1^ [67].

#### 4.3.2. Extraction and Estimation of Anaerobic Metabolism Enzymes

In the process of ADH and ALDH extraction, root tissues were subjected to homogenization using 100 mM HEPES buffer with 2 mM dithiothreitol (with a pH of 6.5). The homogenate was subsequently centrifuged at 10,000× *g* for 15 min at 4 °C. The enzymatic activity of ADH and ALDH was assessed employing the methods reported by Ke et al. [107] and Liu et al. [108].

The extraction of PDC was achieved by subjecting root samples to pulverization in a 50 mM sodium phosphate buffer solution of pH 6.5, supplemented with 5 mM MgCl_2_, 50 µM TPP, and 5 mM β-mercaptoethanol. The resulting homogenate was then subjected to centrifugation at 10,000× *g* for 15 min at 4 °C, as described by Lee and Langston-Unkefer [109]. The assay mixture utilized for analysis consisted of 100 mM sodium phosphate buffer with a pH of 6.5, along with 0.5 µmole of MgCl_2_, 0.35 µmole of TPP, 0.15 µmole of NADH, and 10 units of ADH from Sigma Chemicals Company per ml of the assay solution [110]. The coupled oxidation of NADH was then continuously monitored at 340 nm at 30-sec intervals for a duration of 3 min.

### 4.4. Extraction and Estimation of Metabolites

#### 4.4.1. Extraction and Estimation of Total Glutathione and Reduced Ascorbic Acid Content

In this experimental procedure, leaf tissues weighing 0.2 g were crushed and homogenized in 1.5 mL of 5% chilled metaphosphoric acid. Subsequently, centrifugation was carried out at 10,000× *g* for 10 min. The resulting clear supernatant was utilized for the quantification of ascorbic acid on the basis of its oxidation to dehydroascorbic acid induced by Fe^3+^. The generated ferrous ions Fe (II)) reacted with 2,2′-bipyridyl, producing a red-colored chromogen with a maximum absorption peak at 530 nm, as reported by Singla et al. [111]. Additionally, dehydroascorbate (DHA) content was determined by its reaction with 2,4-dinitrophenylhydrazine (DNPH) in the presence of 85% sulfuric acid, resulting in the formation of an orange-red product. This product was quantified spectrophotometrically at 540 nm, as described by Roe and Oesterling in 1944 [112].

For the analysis of total glutathione, 0.1 g of each tissue was crushed with 2 mL of 5% sulfosalicylic acid, followed by centrifugation at 10,000× *g* for 10 min. A 0.5 mL aliquot of the supernatant was neutralized using 0.75 mL of 0.5 M potassium phosphate buffer of pH 7.5 and employed for total glutathione assay. Another 1 mL aliquot of the supernatant was neutralized with 1.5 mL of 0.5 M potassium phosphate buffer (pH 7.5) and 0.2 mL of 2-vinylpyridine. This mixture was used to assay oxidized glutathione (GSSG) after incubation for 1 h at 25 °C, following the protocol reported by Singla et al. [111].

#### 4.4.2. Extraction and Estimation of H_2_O_2_

Leaf tissue weighing 0.3 g was subjected to homogenization in 2 mL of ice-cold sodium phosphate buffer of pH 7.0. Subsequently, the resulting homogenate was centrifuged at a speed of 10,000× *g* for a duration of 20 min. The quantification of hydrogen peroxide (H_2_O_2_) content was performed in the resulting supernatant [113].

#### 4.4.3. Extraction and Estimation of Chlorophyll Content

For the estimation of chlorophyll content, finely chopped fresh leaf tissues were immersed in a test tube containing 5 mL of dimethyl sulfoxide (DMSO). Subsequently, the mixture was incubated at a temperature of 60 °C for a duration of 2 h. The absorbance of the solution was measured spectrophotometrically at wavelengths of 645 nm and 665 nm, as described by Hiscox and Israelstam [114].

#### 4.4.4. Extraction and Estimation of MDA Content

Malondialdehyde (MDA) quantification was conducted through the application of a thiobarbituric acid reaction, following the method described by Heath and Packer in 1968 [115]. Tissue samples (200 mg) were homogenized in 2 mL of 5% (*w/v*) trichloroacetic acid (TCA) and subsequently subjected to centrifugation at 10,000× *g* for 15 min at room temperature. The resulting supernatant, used for MDA estimation, was combined with an equal volume of 20% (*w/v*) TCA containing 0.5% thiobarbituric acid (TBA). This mixture was then heated at 95 °C for 30 min followed by cooling in an ice bath, and subsequently centrifuged at 10,000× *g* for 10 min. The absorbance of the supernatant was measured at 532 nm and adjusted for non-specific turbidity by subtracting the absorbance at 600 nm. MDA concentrations were determined using an extinction coefficient of 155 mM^−1^cm^−1^. The results were reported as nanomoles of MDA per gram of fresh weight (nmol MDA g^−1^ FW).

### 4.5. Extraction and Estimation of Proline

The proline concentration was quantified utilizing the methodology described by Bates et al. [116]. Following extraction in a 3% *w/v* solution of sulfosalicylic acid, the samples were subjected to centrifugation at 6000× *g* for 15 min. The assay mixture comprised the extract, 2 mL of glacial acetic acid, and 2 mL of acidic ninhydrin solution. Subsequent to heating in boiling water at 100 °C for 1 h, 4 mL of toluene was introduced into the tubes post-cooling, followed by vigorous shaking to ensure thorough mixing. The toluene phase, containing the chromophore, was separated from the aqueous phase, and the absorbance was measured at 520 nm using a spectrophotometer. Proline content was calculated using a standard curve and expressed as mg g^−1^ FW.

### 4.6. Statistical Analysis

All results are expressed as mean ± S.E. (standard error). Version 2016 of Microsoft Excel was used to draw all the graphs. A one-way analysis of variance (ANOVA) followed by the Fisher least significant difference (LSD) test at *p ≤* 0.05 using the statistical software R (version 4.13) was used to determine the statistical significance of the treatment differences. Principal component analysis was also estimated using the same statistical program.

## 5. Conclusions

The assessed sesame genotypes exhibit distinctive genetic diversity, as evidenced by their capacity to endure waterlogged conditions during the early stages of vegetative growth. Out of 142 genotypes tested, 2 genotypes (JLT-8 and GP-70) exhibited remarkable tolerance against waterlogging stress, 5 genotypes were found to be the most susceptible, and 135 genotypes were screened as moderately tolerant/susceptible against waterlogging stress. In-depth morphological and biochemical analysis of these divergent genotypes, specifically the highly tolerant and highly susceptible genotypes, under varying waterlogging durations (0, 24, 48, 72 and 96 h of waterlogging), revealed compelling insights. The tolerant genotypes displayed superior antioxidant and fermentation systems, evidenced by elevated activities and contents of pertinent enzymes and metabolites. This phenomenon persisted across all stress conditions, elucidating the plausible biochemical mechanisms behind their waterlogging tolerance. The seedling parameters were negatively affected by waterlogging in both genotypes; however, the damage was markedly severe in susceptible genotypes. Tolerant genotypes also exhibited higher WTCs. Photosynthetic efficiency was adversely impacted in both genotypes after 48 h of stress, albeit with a higher severity of damage in susceptible genotypes. Adventitious root formation was also observed in both the genotypes as morphological adaption to low oxygen availability. Both genotypes induced the proline content in order to maintain osmotic balance under stress conditions. Consequently, the robust antioxidant potential and efficient anaerobic metabolism observed in the tolerant genotypes serve as key mechanisms enabling their resilience to short-term waterlogging exposure. These findings underscore the promising potential of specific sesame genotypes in enhancing crop resilience against waterlogging stress, offering valuable insights for agricultural practices and breeding programs.

## Figures and Tables

**Table 1:** Eigen value and variability percentage of sesame genotypes for different traits under optimal and stress conditions.

Principal Components	Optimal Condition			Waterlogging Stress		
	Eigen Value	Variance (%)	Cumulative Variance (%)	Eigen Value	Variance (%)	Cumulative Variance (%)
PC1	12.55	39.21	39.21	16.62	51.94	51.94
PC2	7.81	24.41	63.62	7.57	23.67	75.62
PC3	4.59	14.35	77.97	2.59	8.11	83.74
PC4	2.70	8.46	86.43	1.50	4.70	88.44
PC5	1.26	3.95	90.38	0.96	3.00	91.43

**Table 2:** Screening of sesame genotypes for 7 days waterlogging stress at seedling stage.

S.No.	Genotype Name	Source of GERMPLASM	Response to Waterlogging	No. of Genotypes Identified
	JLT-8	ORS ^#^, Jalgaon, MH	Highly Tolerant *	02
	GP-70	ORS, Mauranipur	Highly Tolerant	
	S-0481	AICRP ^##^ Sesame, Jabalpur	Moderately tolerant **	66
	ES-75	AICRP Sesame, Jabalpur	Moderately tolerant	
	S-0273	AICRP Sesame, Jabalpur	Moderately tolerant	
	Tilo/Hana	AICRP Sesame, Jabalpur	Moderately tolerant	
	S-003116	AICRP Sesame, Jabalpur	Moderately tolerant	
	IS-653-A	AICRP Sesame, Jabalpur	Moderately tolerant	
	NIC-17477-I	AICRP Sesame, Jabalpur	Moderately tolerant	
	12-Jun	AICRP Sesame, Jabalpur	Moderately tolerant	
	S-0449	AICRP Sesame, Jabalpur	Moderately tolerant	
	NIL-16426	AICRP Sesame, Jabalpur	Moderately tolerant	
	S-0539	AICRP Sesame, Jabalpur	Moderately tolerant	
	SI-1687-I	AICRP Sesame, Jabalpur	Moderately tolerant	
	NCR/82/No/Bo/NS	AICRP Sesame, Jabalpur	Moderately tolerant	
	NIC-8062	AICRP Sesame, Jabalpur	Moderately tolerant	
	SI-1925	AICRP Sesame, Jabalpur	Moderately tolerant	
	IS-77	AICRP Sesame, Jabalpur	Moderately tolerant	
	S-0627	AICRP Sesame, Jabalpur	Moderately tolerant	
	IS-99-A	AICRP Sesame, Jabalpur	Moderately tolerant	
	EC-334967	AICRP Sesame, Jabalpur	Moderately tolerant	
	SI-3315-16	AICRP Sesame, Jabalpur	Moderately tolerant	
	NIC-7982	AICRP Sesame, Jabalpur	Moderately tolerant	
	IS-446-1-84	AICRP Sesame, Jabalpur	Moderately tolerant	
	EC-310455	AICRP Sesame, Jabalpur	Moderately tolerant	
	SI-3114	AICRP Sesame, Jabalpur	Moderately tolerant	
	EC-334969	AICRP Sesame, Jabalpur	Moderately tolerant	
	EC-334995-I	AICRP Sesame, Jabalpur	Moderately tolerant	
	ES-64	AICRP Sesame, Jabalpur	Moderately tolerant	
	IS-564	AICRP Sesame, Jabalpur	Moderately tolerant	
	S-0210	AICRP Sesame, Jabalpur	Moderately tolerant	
	IS-350	AICRP Sesame, Jabalpur	Moderately tolerant	
	NIC-8343	AICRP Sesame, Jabalpur	Moderately tolerant	
	GRT-8359	AICRP Sesame, Jabalpur	Moderately tolerant	
	S-0281	AICRP Sesame, Jabalpur	Moderately tolerant	
	IS-712	AICRP Sesame, Jabalpur	Moderately tolerant	
	GRT-83128	AICRP Sesame, Jabalpur	Moderately tolerant	
	NIC-16218	AICRP Sesame, Jabalpur	Moderately tolerant	
	KJS-21	AICRP Sesame, Jabalpur	Moderately tolerant	
	S-0268-C	AICRP Sesame, Jabalpur	Moderately tolerant	
	SI-1865-1-B	AICRP Sesame, Jabalpur	Moderately tolerant	
	IS-346	AICRP Sesame, Jabalpur	Moderately tolerant	
	ES-742-B	AICRP Sesame, Jabalpur	Moderately tolerant	
	GRT-8630-C	AICRP Sesame, Jabalpur	Moderately tolerant	
	ES-141-1-84-C	AICRP Sesame, Jabalpur	Moderately tolerant	
	OMT-4	AICRP Sesame, Jabalpur	Moderately tolerant	
	Belatal Local	AICRP Sesame, Jabalpur	Moderately tolerant	
	IS-52	AICRP Sesame, Jabalpur	Moderately tolerant	
	IS-552	AICRP Sesame, Jabalpur	Moderately tolerant	
	IS-178-C	AICRP Sesame, Jabalpur	Moderately tolerant	
	Sel-07-2	ORS, Jalgaon, MH	Moderately tolerant	
	NIC 8401	ORS, Jalgaon, MH	Moderately tolerant	
	JT-11	ORS, Jalgaon, MH	Moderately tolerant	
	Thilrani	ORS, Jalgaon, MH	Moderately tolerant	
	KMR-24	ORS, Jalgaon, MH	Moderately tolerant	
	JLS-1392-2	ORS, Jalgaon, MH	Moderately tolerant	
	RT-127	ORS, Jalgaon, MH	Moderately tolerant	
	LT-5	ORS, Jalgaon, MH	Moderately tolerant	
	Prachi	ORS, Jalgaon, MH	Moderately tolerant	
	HT 1	Cultivar	Moderately tolerant	
	GT-5	Cultivar	Moderately tolerant	
	MT-10-23-3	ORS, Mauranipur	Moderately tolerant	
	MT-10-8-1	ORS, Mauranipur	Moderately tolerant	
	MT-8-04	ORS, Mauranipur	Moderately tolerant	
	GP-79	ORS, Mauranipur	Moderately tolerant	
	GP-4	ORS, Mauranipur	Moderately tolerant	
	GP-63	ORS, Mauranipur	Moderately tolerant	
	TIC-74	AICRP Sesame, Jabalpur	Susceptible ^$^	69
	Anand Local	AICRP Sesame, Jabalpur	Susceptible	
	IS-722-2-84-I	AICRP Sesame, Jabalpur	Susceptible	
	S-0606	AICRP Sesame, Jabalpur	Susceptible	
	G-10	AICRP Sesame, Jabalpur	Susceptible	
	Oct-81	AICRP Sesame, Jabalpur	Susceptible	
	EC-182832	AICRP Sesame, Jabalpur	Susceptible	
	G-18	AICRP Sesame, Jabalpur	Susceptible	
	NIC-8282	AICRP Sesame, Jabalpur	Susceptible	
	IS-387	AICRP Sesame, Jabalpur	Susceptible	
	IS-205-I	AICRP Sesame, Jabalpur	Susceptible	
	NIC-9835	AICRP Sesame, Jabalpur	Susceptible	
	ES-110-A	AICRP Sesame, Jabalpur	Susceptible	
	S-0619	AICRP Sesame, Jabalpur	Susceptible	
	S-0069	AICRP Sesame, Jabalpur	Susceptible	
	ES-139-2-84	AICRP Sesame, Jabalpur	Susceptible	
	IC-152485	AICRP Sesame, Jabalpur	Susceptible	
	SI-3275	AICRP Sesame, Jabalpur	Susceptible	
	IS-436-3-84	AICRP Sesame, Jabalpur	Susceptible	
	IS-449	AICRP Sesame, Jabalpur	Susceptible	
	EC-334987	AICRP Sesame, Jabalpur	Susceptible	
	IS-641-2-84	AICRP Sesame, Jabalpur	Susceptible	
	IC-2621694	AICRP Sesame, Jabalpur	Susceptible	
	G-19	AICRP Sesame, Jabalpur	Susceptible	
	EC-204704	AICRP Sesame, Jabalpur	Susceptible	
	IS-90	AICRP Sesame, Jabalpur	Susceptible	
	I-68	AICRP Sesame, Jabalpur	Susceptible	
	IS-686	AICRP Sesame, Jabalpur	Susceptible	
	TMV-12-52	AICRP Sesame, Jabalpur	Susceptible	
	KMR-48	AICRP Sesame, Jabalpur	Susceptible	
	GRT-83138	AICRP Sesame, Jabalpur	Susceptible	
	S-0223	AICRP Sesame, Jabalpur	Susceptible	
	IS-62-I	AICRP Sesame, Jabalpur	Susceptible	
	EC-303423-C	AICRP Sesame, Jabalpur	Susceptible	
	S-0403	AICRP Sesame, Jabalpur	Susceptible	
	Juland Sahame	AICRP Sesame, Jabalpur	Susceptible	
	GSM-21	AICRP Sesame, Jabalpur	Susceptible	
	NIC-17362-A	AICRP Sesame, Jabalpur	Susceptible	
	847-1-C	AICRP Sesame, Jabalpur	Susceptible	
	GRT-8336	AICRP Sesame, Jabalpur	Susceptible	
	SI-3075	AICRP Sesame, Jabalpur	Susceptible	
	SI-2670	AICRP Sesame, Jabalpur	Susceptible	
	IS-151-B	AICRP Sesame, Jabalpur	Susceptible	
	IS-8480-B	AICRP Sesame, Jabalpur	Susceptible	
	SI-1248-B	AICRP Sesame, Jabalpur	Susceptible	
	IS-646-3-84-C	AICRP Sesame, Jabalpur	Susceptible	
	RT-54	ORS, Jalgaon, MH	Susceptible	
	Uma	ORS, Jalgaon, MH	Susceptible	
	RT-283	ORS, Jalgaon, MH	Susceptible	
	EC 377015	ORS, Jalgaon, MH	Susceptible	
	EC 370840	ORS, Jalgaon, MH	Susceptible	
	IS-1162	ORS, Jalgaon, MH	Susceptible	
	Pragati (MT 75)	Cultivar	Susceptible	
	RT 351	Cultivar	Susceptible	
	HT 2	Cultivar	Susceptible	
	GT-6	Cultivar	Susceptible	
	GT-10	Cultivar	Susceptible	
	Local Rath	ORS, Mauranipur	Susceptible	
	Local Material-7	ORS, Mauranipur	Susceptible	
	GP-174	ORS, Mauranipur	Susceptible	
	GP-179	ORS, Mauranipur	Susceptible	
	GP-177	ORS, Mauranipur	Susceptible	
	GP-182	ORS, Mauranipur	Susceptible	
	GP-164	ORS, Mauranipur	Susceptible	
	GP-18	ORS, Mauranipur	Susceptible	
	GP-153	ORS, Mauranipur	Susceptible	
	GP-80	ORS, Mauranipur	Susceptible	
	GP-190	ORS, Mauranipur	Susceptible	
	GP-152	ORS, Mauranipur	Susceptible	
	GP-140	ORS, Mauranipur	Susceptible	
	NIC-161848	AICRP Sesame, Jabalpur	Highly Susceptible ^$$^	05
	EC-335003	AICRP Sesame, Jabalpur	Highly Susceptible	
	RJS-Bo	AICRP Sesame, Jabalpur	Highly Susceptible	
	TKG-306	ORS, Jalgaon, MH	Highly Susceptible	
	R-III-F6	ORS, Mauranipur	Highly Susceptible	

* 100% survival of plants after stress treatment. Among symptoms, only leaf yellowing appeared after 2nd day of stress and remained moderate till the end of experiment, however no leaf browning, wilting, drooping, or necrosis was observed till end of the experiment; ** 100% survival of plants after stress treatment. Leaf yellowing appeared after 2nd day and become severe till the end of experiment. Leaf browning was observed after 4th day and remained moderate till end of the experiment. Leaf wilting was observed after day 6th of stress and remained mild till end of the experiment. No leaf drooping or necrosis were observed till end of the experiment; **^$^** 60–80% survival of plants at the end of the stress treatment. Leaf yellowing appeared after 1st day and become severe till the end of experiment. Leaf browning appeared after 3rd day and become severe till the end of the experiment. Leaf wilting was observed after day 5th day of stress exposure and remained moderate till end of the experiment. Leaf drooping was observed after day 5th day of the stress imposition and remained mild till end of the experiment. Necrosis and mortality were observed in few plants (20–40%) at the end of the experiment; ^$$^ 20–40% survival of plants at the end of the stress treatment. Leaf yellowing appeared after 1st day and become severe till the end of experiment. Leaf browning was observed after 3rd day and become severe till the end of experiment. Leaf wilting was observed after day 4th of stress and become severe till the end of experiment. Leaf drooping was observed after day 5th of stress and become severe till the end of experiment. Necrosis and mortality were observed in most of the plants (60–80%) at the end of the experiment; ^#^ Oilseed research station; ^##^ All India Coordinated research centre.

## Data Availability

The data are contained within the article.
